# The noncoding RNA BC200 associates with polysomes to positively regulate mRNA translation in tumor cells

**DOI:** 10.1074/jbc.RA120.015775

**Published:** 2020-11-24

**Authors:** Evan P. Booy, Daniel Gussakovsky, Taegi Choi, Sean A. McKenna

**Affiliations:** Department of Chemistry, University of Manitoba, Winnipeg, Manitoba, Canada

**Keywords:** BC200, brain cytoplasmic RNA 1 (BCYRN1), translation regulation, Alu, long noncoding RNA (long ncRNA, lncRNA), ribosome, RNA interference (RNAi), RNA–protein interaction, breast cancer, BCMUT, GapmeR resistant mutant of BC200, LNA, locked nucleic acid, SINE, short interspersed nuclear elements, TO1-biotin, thiazole orange biotin

## Abstract

BC200 is a noncoding RNA elevated in a broad spectrum of tumor cells that is critical for cell viability, invasion, and migration. Overexpression studies have implicated BC200 and the rodent analog BC1 as negative regulators of translation in both cell-based and *in vitro* translation assays. Although these studies are consistent, they have not been confirmed in knockdown studies and direct evidence for this function is lacking. Herein, we have demonstrated that BC200 knockdown is correlated with a decrease in global translation rates. As this conflicts with the hypothesis that BC200 is a translational suppressor, we overexpressed BC200 by transfection of *in vitro* transcribed RNA and transient expression from transfected plasmids. In this context BC200 suppressed translation; however, an innate immune response confounded the data. To overcome this, breast cancer cells stably overexpressing BC200 and various control RNAs were developed by selection for genomic incorporation of a plasmid coexpressing BC200 and the neomycin resistance gene. Stable overexpression of BC200 was associated with elevated translation levels in pooled stable cell lines and isolated single-cell clones. Cross-linking sucrose density gradient centrifugation demonstrated an association of BC200 and its reported binding partners SRP9/14, CSDE1, DHX36, and PABPC1 with both ribosomal subunits and polysomal RNA, an association not previously observed owing to the labile nature of the interactions. In summary, these data present a novel understanding of BC200 function as well as optimized methodology that has far reaching implications in the study of noncoding RNAs, particularly within the context of translational regulatory mechanisms.

Alu elements are primate-specific abundant short interspersed nuclear elements (SINEs) that are present in humans in excess of 1 million copies and comprise 10.7% of the human genome (1). Although few Alu elements are capable of retrotransposition, a large number are transcriptionally active (2, 3). The prevalence of cellular Alu RNA is primarily due to insertions within the 5′ and 3′ untranslated regions and introns of mRNAs. In addition to transcription as part of RNA polymerase II transcribed mRNAs, Alu elements are themselves transcribed *via* recruitment of RNA polymerase III to internal promoter sequences of the Alu repeat itself (4). The pervasiveness and sequence similarity between various Alu elements add a considerable research challenge to the study of these expressed SINEs (1). Nonetheless, expressed Alu elements have been reported to influence diverse cellular processes such as gene transcription, RNA splicing, RNA localization, and RNA editing (1, 4, 5).

BC200 (Brain cytoplasmic RNA 1, BCYRN1) is a 200-nt anthropoid primate-specific monomeric Alu RNA that is abundantly expressed in the brain (6, 7, 8, 9). In a similar manner to its rodent counterpart BC1, BC200 is postulated to regulate localized translation in neuronal dendrites (10, 11). BC1 is a 143-nt transcribed ID element, a type of SINE found in variable numbers of copies among rodent species (12). BC1 exhibits a highly similar expression profile to BC200 and is thought to fulfill an analogous function despite being unique in sequence and origin (13, 14). In prosimian primates, a dimeric Alu insertion is found at the identical location on chromosome 2 as BC200 (15). This RNA has been termed G22 and is a 335-nt RNA that displays a similar expression pattern as BC200, suggesting a functional overlap between these RNAs in distinct species (15, 16).

Although BC200, BC1, and G22 RNAs are derived from SINE retrotransposons and exhibit nearly identical expression patterns, they diverge in sequence considerably. BC200 comprises a 5′ left Alu-J monomer (nucleotides 1–120) followed by a central 40-nt adenosine-rich stretch and a 3′ region that contains 25 nt of unique sequence as well as a consecutive run of 12 cytosines prior to the transcriptional termination sequence (6, 9, 17). BC1 exhibits little sequence similarity to BC200 with the exception of an adenosine-rich stretch that spans approximately 50 nucleotides (13). G22 on the other hand is a dimeric Alu that has only a short 9-nt stretch of adenosines prior to a unique and C-rich 3′ end that bears similarity to BC200 (16).

Although BC200 expression is normally restricted to the brain and to a lesser extent testes and ovaries, it is also elevated in several tumor types compared with normal matched tissues (7, 18, 19, 20, 21, 22, 23). In tumor cell line models, BC200 is critical for cell viability as well as to promote cell migration and invasion (7, 24, 25, 26, 27). In terms of specific function, overexpression studies of both BC200 and BC1 have suggested a role for both RNAs in negative regulation of translation in both in-cell as well as *in vitro* translation assays (28, 29, 30, 31, 32, 33). Supporting these reports, we have previously described the interaction of BC200 with a number of proteins that implicate potential roles in mRNA stability, translation, and splicing (CSDE1, DHX36, PABPC1, PABPN1, HNRNPK, SRP9/14, SYNCRIP) (17, 34). Reinforcing a probable common function with BC200, the G22 RNA shares a number of key protein binding partners with BC200 including SRP9/14 and PABPC1 (16).

Alu RNAs are present at relatively low levels in human cells but are upregulated by various cellular stresses including translation arrest, heat shock, and viral infection (35, 36, 37, 38). Translational inhibitors such as cycloheximide result in a rapid and dramatic upregulation of the expression of Alu RNA (35). Alu RNAs have been shown in a number of contexts to increase mRNA translation rates (33, 35, 39, 40, 41), and the expression of Alu RNAs is correlated with mRNA translation rates (35, 39). Although Alu RNAs can enhance translation, their role is still unclear. Alu RNA in complex with SRP9/14 has been shown to inhibit translation by *in vitro* translation assays, whereas supplementation of Alu RNA alone stimulated translation (33). Furthermore, Alu RNAs inhibit the activity of EIF2AK2 (PKR) under cell stress, providing a possible mechanism by which they may act to increase translation rates in this context (41).

In previous studies, Alu RNA did not show an association with either free ribosomal subunits, monosomes (a single intact ribosome), or polysomes (actively translating ribosomes on a single mRNA) in sucrose density gradients and were proposed to act at the initiation stage of translation and not affect elongation rates (33). In agreement with this, both BC200 and other transcribed Alu RNAs have been shown to exist as an ∼11S ribonucleoprotein particle, which sediments considerably slower than the small 40S ribosomal subunit (33, 42, 43).

In the present study we observed a significant reduction in global translation rates upon BC200 knockdown using locked nucleic acid (LNA) GapmeRs in a panel of tumor cell lines. MCF-7 cells were selected for further study as they exhibit elevated BC200 expression, rely on BC200 for cell viability, and have been used to assess the protein components of the BC200 RNP (7, 34). BC200 has previously been reported as a negative regulator of translation; however, these studies involved either transfection of *in vitro* transcribed RNA (30, 31) or the use of non-primate-based *in vitro* translation assays (28, 29, 30, 33, 44). Although we were able to replicate these results in-cell by transfection of *in vitro* transcribed BC200, a scrambled control RNA produced a more robust effect. Transfection of all *in vitro* transcribed RNAs tested induced an innate immune response as was detected by induction of RIG-I expression. To avoid the innate immune response elicited by RNA transfection, we overexpressed BC200 from a transiently transfected plasmid; however, control transfections significantly impacted cellular translation rates for as long as 96 h post transfection. To avoid these pitfalls, cells stably expressing BC200 were generated that demonstrated a significant increase in global translation rates in response to elevated BC200. We observed a positive correlation between expression of BC200 and relative translation rates in isolated single-cell clones. Initial sucrose density gradient centrifugation experiments were in agreement with the literature and indicated that BC200 did not comigrate with ribosomal subunits or polysomal RNA; however, in these experiments we also observed a complete separation of BC200 from some of its previously reported binding partners. To investigate this discrepancy, we optimized a cross-linking sucrose density gradient centrifugation protocol that demonstrated comigration of BC200 with published protein binding partners as well as association with polysomal RNA. This study for the first time presents evidence that BC200 is in fact a translational activator and likely exerts its function through interactions with actively translating mRNAs.

## Results

### Knockdown of BC200 reduces global mRNA translation rates

**Figure 1:**
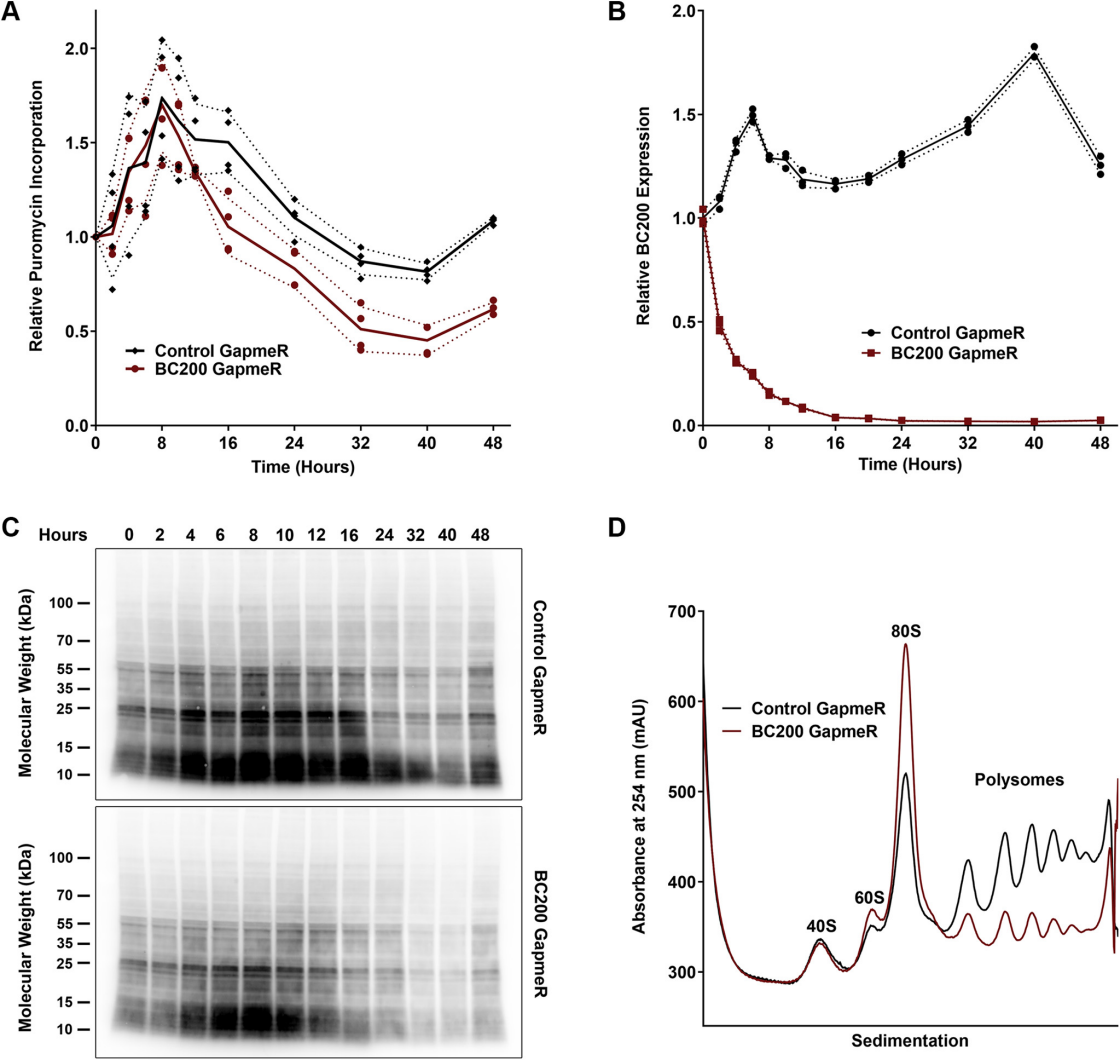
**BC200 knockdown inhibits translation.***A*, quantification of puromycin incorporation by densitometry analysis of Western blots performed with an anti-puromycin antibody following transfection of MCF-7 cells with a control or BC200 targeting LNA GapmeR. Lines represent the mean background corrected sum of lane signal intensity for each of four biological replicates. Standard deviation is represented by the dashed lines. *B*, relative BC200 expression was measured by RT-qPCR on 25 ng of RNA extracted from a fraction of the samples used in (*A*). Lines represent the mean of three biological replicates measured in duplicate. Dashed lines indicate standard deviation. *C*, western blot of puromycin incorporation time course used to generate the data in (*A*). *D*, polysome analysis by sucrose density gradient centrifugation of cell lysates 48 h following transfection of a control (*black*) or BC200 targeting LNA GapmeR (*red*). Profiles are representative of at least three independent replicates.

We have recently reported the interaction of BC200 with a number of protein binding partners that supports the proposed function of BC200 as a regulator of mRNA translation (34). As previous studies looked exclusively at translational changes in response to overexpression of BC200 (28, 29, 30, 31, 32, 33), we sought to assess the impact of BC200 knockdown on mRNA translation rates. Knockdown of BC200 in MCF-7 cells with an LNA GapmeR resulted in a significant reduction in translation rates within 12 h as measured by puromycin incorporation (Fig. 1*A*). Puromycin is an aminonucleoside antibiotic that is incorporated into actively translating peptides causing chain termination, a process that can be monitored using monoclonal antibodies that exhibit high specificity for puromycin (45). The reduction in translation was concurrent with efficient reduction in BC200 expression (Fig. 1, *A–C*). Translation rates remained suppressed through 48 h as compared with cells transfected with a nontargeting control GapmeR.

A second commonly used method to monitor translation rates is polysome profiling. Polysome profiling involves separation of cellular components by density gradient centrifugation allowing for separation of 40S and 60S ribosomal subunits from intact ribosomes (monosomes, 80S) and mRNAs that are being actively translated by multiple ribosomes simultaneously (polysomes). Each subpopulation is clearly identified as well-differentiated peaks in the absorbance trace at 260 nm and the relative quantity of polysomes is considered indicative of global translation rate (46, 47). Polysome profiles performed 48 h post transfection demonstrated a dramatic reduction in polysomal RNA and increase in the monosome fraction consistent with reduced mRNA translation rates (Fig. 1D). Similar results were obtained in SK-BR-3, T-47D, A-375, A549, MDA-MB-231, and HEK-293T cells (Fig. S1, *A*–*G*, *I*). SK-OV-3 cells, which exhibited the lowest expression of BC200 among the tested cell lines, demonstrated a negligible impact of BC200 knockdown on translation (Fig. S1, *H* and *I*).

### Transfection of *in vitro* transcribed RNAs reduces translation and induces RIG-I expression

**Figure 2:**
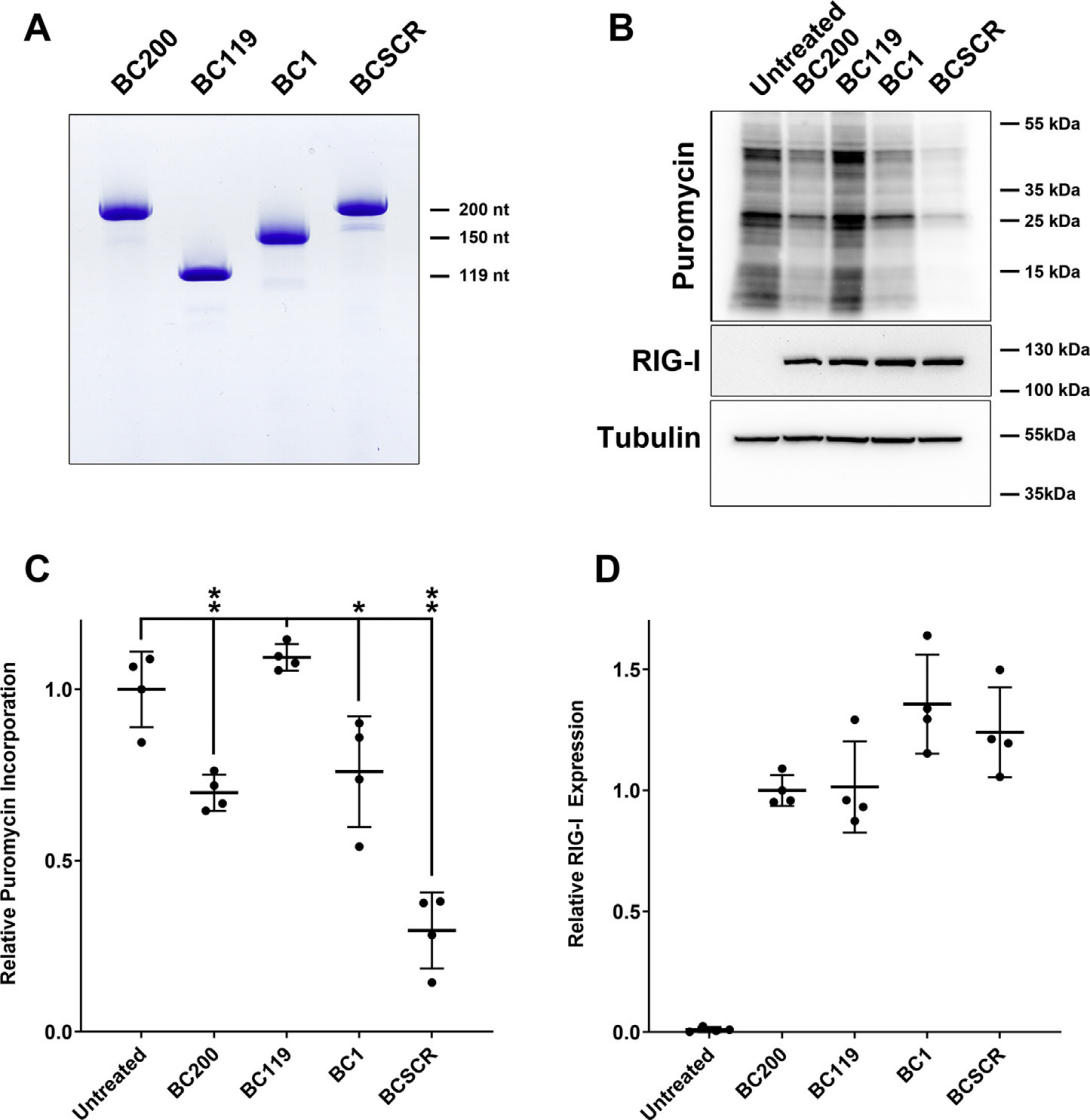
**Transfected *in vitro* transcribed BC200 inhibits translation.***A*, native Tris-Borate EDTA gel electrophoresis of 1 μg each of the indicated purified RNAs stained with Toluidine Blue O. *B*, western blot of protein lysates from cells transfected with the indicated RNAs with antibodies specific to puromycin, RIG-I, and Tubulin. Cell lysates were collected 14 h post transfection. Western blots are representative of three biological replicates. *C*, quantification of puromycin incorporation by densitometry analysis of the Western blots shown in (*B*). Horizontal lines represent the mean background corrected sum of lane signal intensity of four biological replicates ± standard deviation. ∗ indicates *p* < 0.05, ∗∗ indicates *p* < 0.005 by paired two-tailed *t* test. *D*, as in (*C*), quantification of RIG-I signal intensity for the indicated conditions.

**Figure 3:**
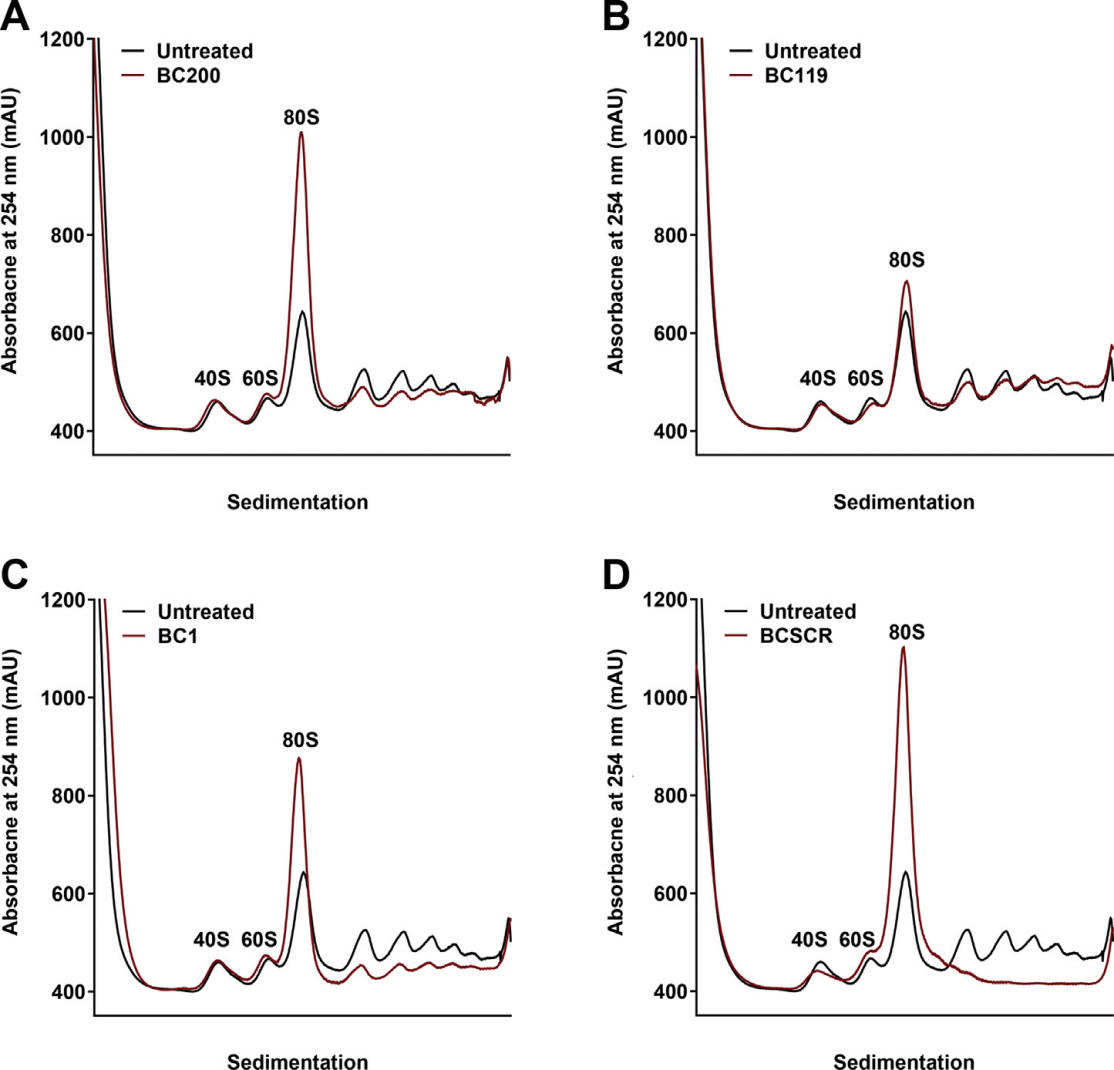
**Transfection of *in vitro* transcribed BC200 shifts the ratio of polysomes to monosomes.***A*, polysome analysis by sucrose density gradient centrifugation of cell lysates 14 h following transfection of an *in vitro* transcribed BC200 RNA (*red*) as compared with untreated cells (*black*). *B*, as in (*A*) for BC119. (*C*) as in (*A*) for BC1. *D*, as in (*A*) for BCSCR.

As knockdown of BC200 suppressed translation and because this result is inconsistent with expectations based on assays performed with *in vitro* transcribed RNAs, we attempted to replicate the BC200 overexpression studies in MCF-7 cells. BC200, a truncation consisting of only the Alu portion of BC200 (BC119), murine BC1, and a scrambled version of the BC200 sequence (BCSCR) were *in vitro* transcribed from plasmid templates and purified (Fig. 2*A*). With the exception of BC119, transfection of these RNAs into MCF-7 cells resulted in a significant reduction in translation rates as measured by puromycin incorporation (Fig. 2, *B* and *C*) and polysome profiles (Fig. 3, *A*–*D*). As we have previously observed an innate immune response from transfection of *in vitro* transcribed RNAs, and as interactions of transfected *in vitro* transcribed BC200 with proteins mediating antiviral response could not be recapitulated in reverse experiments with the endogenous RNA, we suspected this to be an experimental artifact (34). Supporting this, others have also reported that 5′-triphosphate containing *in vitro* transcribed RNAs trigger an innate immune response upon transfection (48). Western blotting of lysates from cells transfected with all four RNAs exhibited a robust induction of RIG-I expression (Fig. 2, *B* and *D*). RIG-I induction in response to foreign nucleic acids is well documented to lead to antiviral signaling cascades that result in translation arrest (49).

### Transient transfection of plasmid DNA causes a prolonged suppression of global translation rates

**Figure 4:**
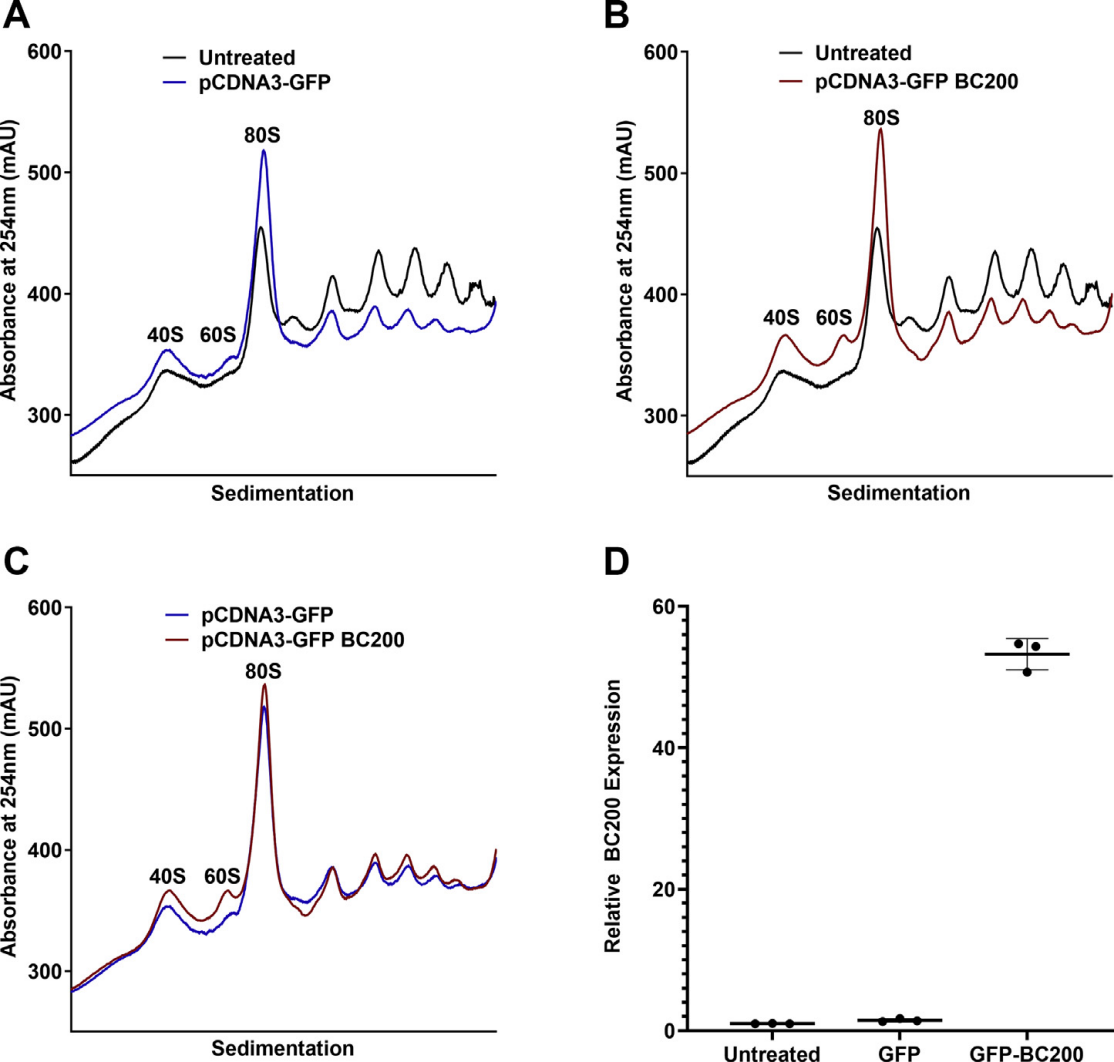
**Transient plasmid transfection impairs mRNA translation.***A*, polysome analysis by sucrose density gradient centrifugation of cell lysates 96 h following transfection of a GFP reporter plasmid (*blue*) as compared with untreated cells (*black*). *B*, as in (*A*), comparison of a BC200 expression plasmid (*red*) with untreated cells (*black*). *C*, overlay of polysome profiles from cells transfected with a GFP reporter plasmid (*blue*) with the same plasmid containing a BC200 expression cassette (*red*). *D*, quantification of BC200 expression by RT-qPCR analysis of DNase I-treated RNA extracted from the samples used in *A–C*. Horizontal line represents the mean of three replicates ± standard deviation.

As results obtained from the transfection of *in vitro* transcribed RNAs were likely obscured by the cellular antiviral response, we attempted to express the RNAs from transfected plasmids as an alternative approach. Plasmid transfection into MCF-7 cells caused a dramatic reduction in polysome levels that persisted 96 h post transfection (Fig. 4*A*). Plasmids expressing GFP alone or a GFP reporter plasmid that also contained a BC200 expression cassette were transfected into MCF-7 cells. The BC200 expression plasmid resulted in an approximately 50-fold increase in relative BC200 expression as measured by RT-qPCR (Fig. 4*D*). Both control and BC200 expression plasmids had translation levels reduced to a nearly indistinguishable extent (Fig. 4, *B* and *C*). As plasmid DNA transfection can, in a similar manner as RNA, illicit an innate immune response, this approach was also considered unsuitable for the study of overexpressed translational regulators (50, 51).

### Stable overexpression of BC200 enhances translation

**Figure 5:**
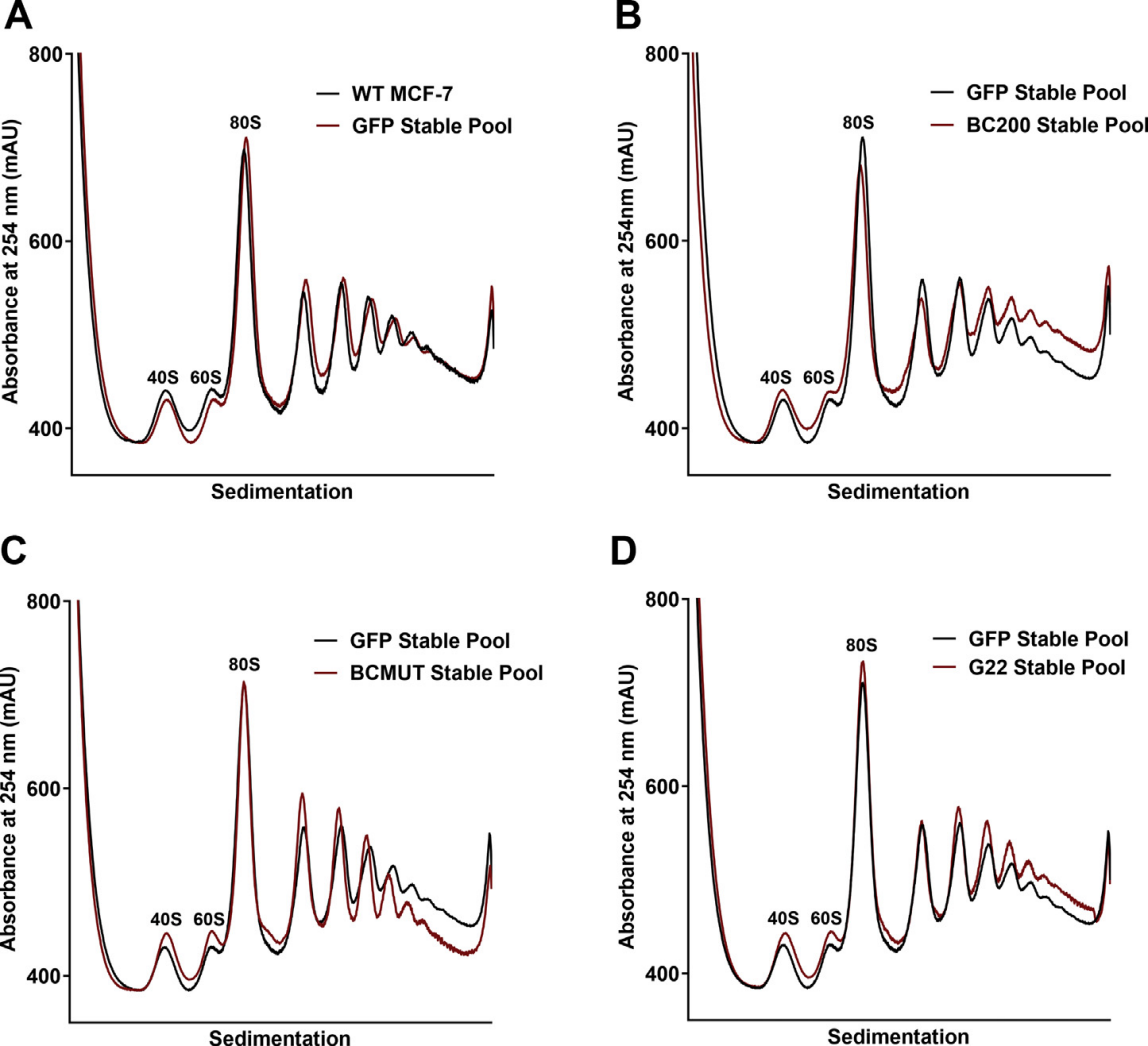
**Stable transfection of BC200 elevates the fraction of polysomal RNA.***A*, polysome analysis by sucrose density gradient centrifugation of cell lysates 48 h following plating 15 × 10^6^ cells into 150-mm dishes. Polysome profile from wild-type MCF-7 cells (*black*) is overlaid with a profile from cells stably selected to express a GFP reporter plasmid (*red*). *B*, as in (*A*), overlaid polysome profiles of cells stably expressing a GFP reporter plasmid (*black*) with cells stably expressing a GFP reporter plasmid with a BC200 expression cassette (*red*). *C*, as in (*B*) with cells stably expressing a GFP reporter plasmid with a BCMUT expression cassette (*red*). *D*, as in (*B*) with cells stably expressing a GFP reporter plasmid with a G22 expression cassette (*red*).

To circumvent the pitfalls associated with transient nucleic acid delivery by transfection, we generated MCF-7 cells that stably incorporated expression plasmids for BC200, a GapmeR resistant mutant of BC200 (BCMUT) as well as the G22 RNA of *Galago moholi*. The rationale for developing cells stably expressing BCMUT and the functionally related G22 RNA from prosimian primates was to rescue the phenotype of B200 knockdown. All plasmids included a GFP reporter gene as well as G418 resistance cassette. Following prolonged selection with G418, relative mRNA translation rates were determined by performing polysome profiles of the stable pooled cells in parallel with wildtype MCF-7. Cells were maintained in media with G418 but cultured in the absence of antibiotics prior to and throughout all experiments. Pooled cells stably expressing the empty GFP reporter plasmid exhibited a polysome profile that superimposed nearly identically with wildtype cells (Fig. 5*A*). This was in contrast to the BC200 expressing stable pool that demonstrated a marked increase in high-order polysomes compared with GFP alone (Fig. 5*B*). The RNAi-resistant BC200 mutant demonstrated the opposite result with a marked decrease in higher-order polysomes (Fig. 5*C*), indicating that the region mutated is necessary for function and BCMUT may be acting in a dominant negative manner. The G22 overexpressing cells displayed an elevation in higher order polysomes albeit to a lesser degree as was observed for BC200 (Fig. 5*D*).

**Figure 6:**
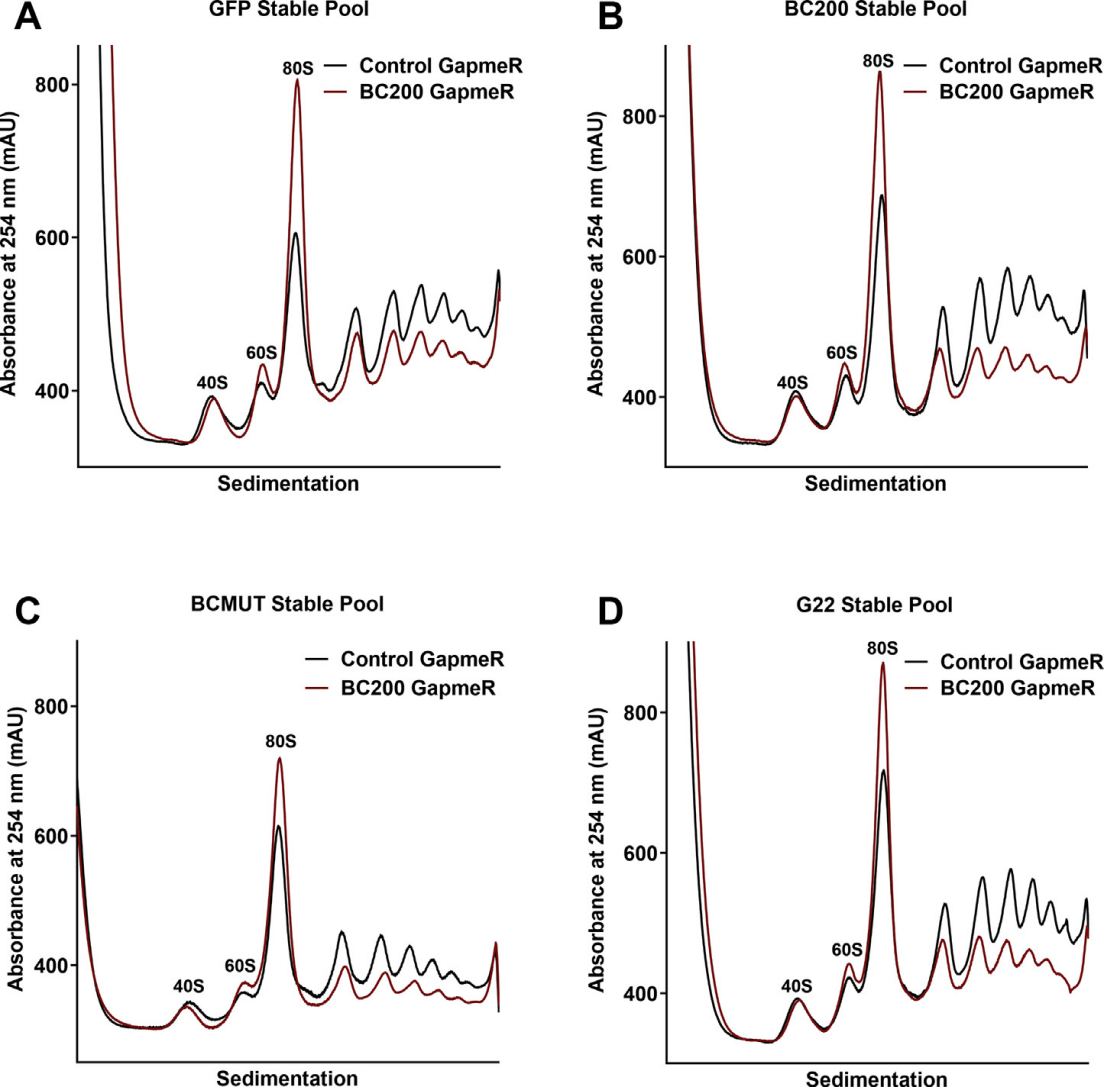
**BCMUT and G22 expression cannot compensate for loss of BC200 expression.***A*, polysome analysis by sucrose density gradient centrifugation of cell lysates 48 h following transfection of a nontargeting control GapmeR (*black*) as compared with a BC200 targeting GapmeR (*red*) in cells stably expressing a GFP reporter plasmid. *B*, as in (*A*), with cells stably expressing a GFP reporter plasmid with a BC200 expression cassette (*red*). *C*, as in (*A*) with cells stably expressing a GFP reporter plasmid with a BCMUT expression cassette (*red*). *D*, as in (*A*) with cells stably expressing a GFP reporter plasmid with a G22 expression cassette (*red*).

To determine if BCMUT or G22 could compensate for BC200, all four stable pooled cell lines were transfected with either nontargeting (control) LNA GapmeR or an LNA GapmeR targeting the 3′ unique sequence of BC200, and polysome profiles were performed 48 h post transfection. In all cell lines a distinct reduction in higher-order polysomes was observed upon BC200 knockdown and no appreciable rescue of the knockdown phenotype was observed. (Fig. 6, *A*–*D*).

**Figure 7:**
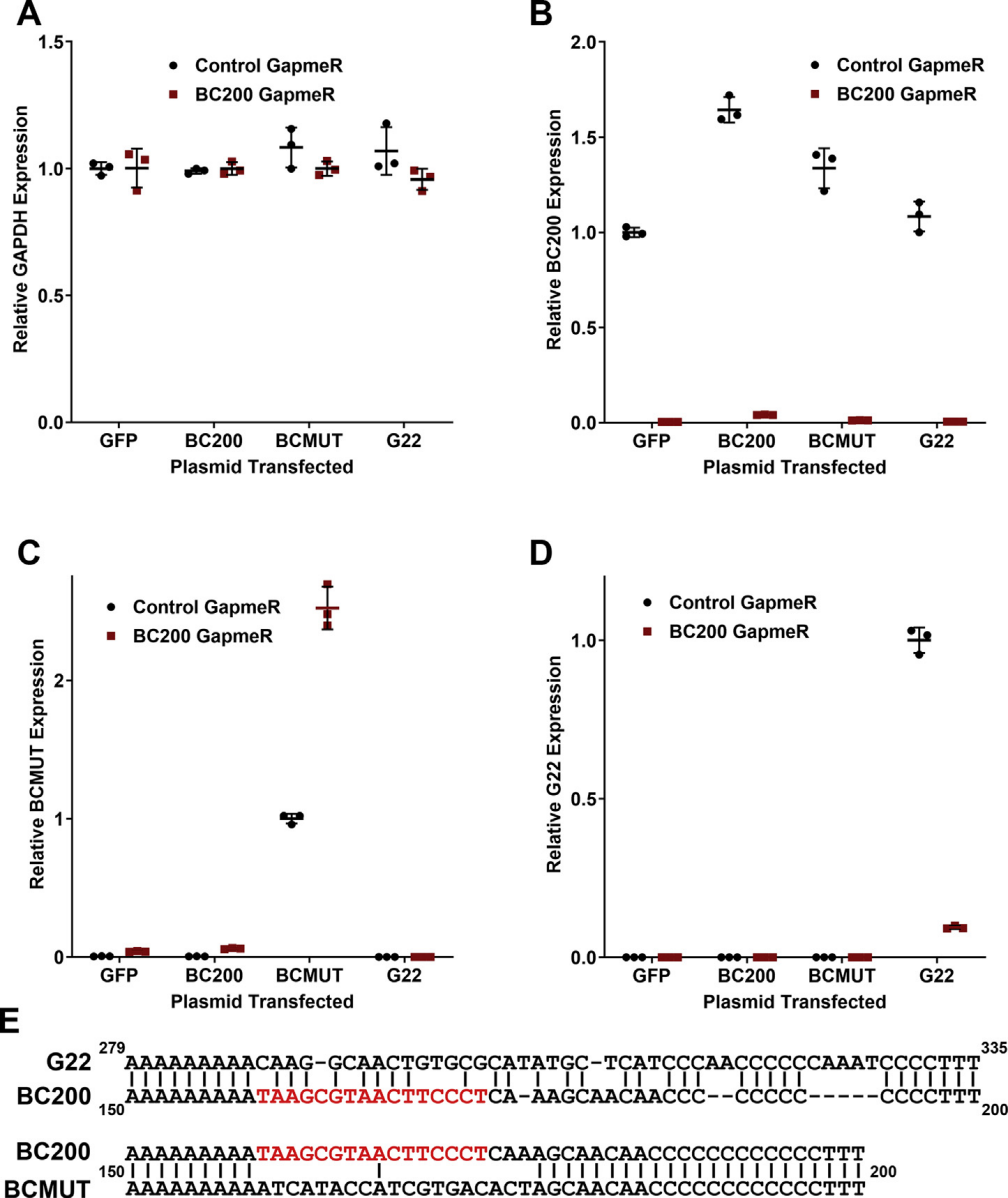
**BC200 knockdown is efficient in stable pooled cells and causes an elevation in BCMUT expression and decrease in the G22 RNA.***A*, RT-qPCR analysis of relative GAPDH expression 48 h following transfection of a nontargeting control GapmeR (*black circles*) as compared with a BC200 targeting GapmeR (*red squares*) in cells stably expressing the indicated plasmids. Horizontal line represents the mean of samples measured in triplicate ± standard deviation. *B*, as in (*A*) analyzing BC200 expression by RT-qPCR. *C*, As in (*A*) analyzing BCMUT expression by RT-qPCR. *D*, as in (*A*) analyzing G22 expression by RT-qPCR. *E*, sequence alignment of the 3′ end of the *Galago moholi* G22 RNA (nucleotides 279–335) with the BC200 RNA 3′ end (nucleotides 150–200) and alignment of the BC200 RNA with the RNAi resistant mutant (BCMUT).

Investigation of RNA expression under these conditions revealed that the house-keeping gene GAPDH exhibited nearly identical expression between the cell lines (Fig. 7*A*). BC200 expression was elevated in the BC200 overexpressing pool as expected, however, to considerably a lesser degree as was observed in transient transfections (Fig. 7*B*, Fig. 4*D*). An increase in BC200 was also observed in the BCMUT, and marginally in the G22 overexpressing pools (Fig. 7*B*). BC200 knockdown was efficient in all of cell lines tested (Fig. 7*B*), and also resulted in a significant upregulation in the expression of BCMUT (Fig. 7*C*). Unexpectedly, the BC200 targeting GapmeR brought about a substantial reduction in G22 expression (Fig. 7*D*), possibly due to sequence similarity in the 3′ end of the RNA (Fig. 7*E*) or an indirect regulatory response of RNA polymerase III–mediated Alu RNA transcription mediated by BC200 knockdown. Regardless of the mechanism, neither BCMUT nor G22 was suitable to rescue the BC200 knockdown phenotype. Although BCMUT is unable to rescue the phenotype, the fact that it appears to be acting in a dominant negative manner to repress translation supports the conclusion that BC200 acts as a translational activator.

**Figure 8:**
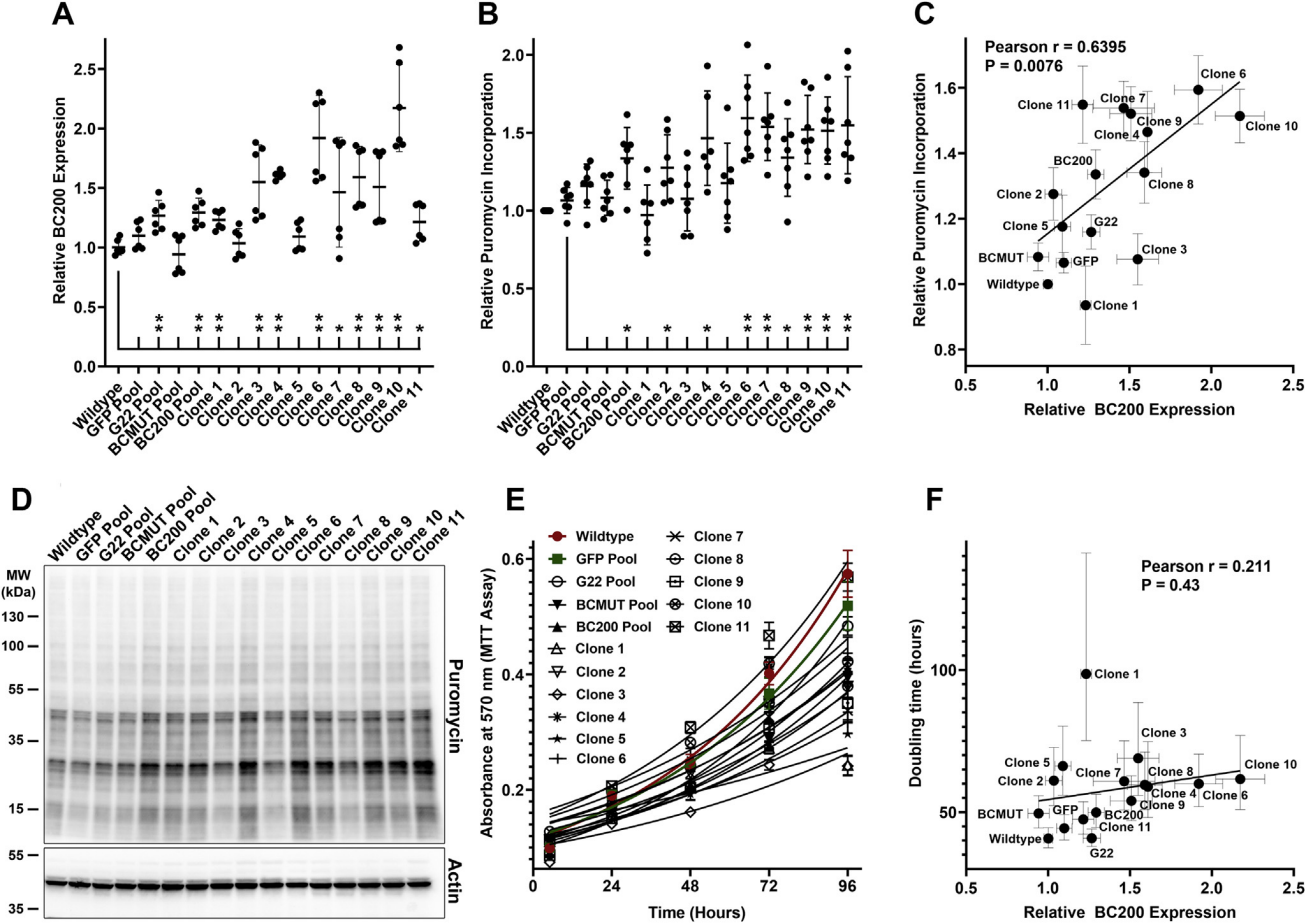
**BC200 expression correlates with elevated translation rates and stable clones exhibit a reduction in cell growth rate.***A*, relative BC200 expression was measured by RT-qPCR on 25 ng of RNA extracted from each of the indicated cell lines. Data represent the average of two RNA samples per cell line measured in triplicate ± standard deviation. ∗ indicates *p* < 0.05, ∗∗ indicates *p* < 0.005 by paired two-tailed *t* test. *B*, quantification of puromycin incorporation by densitometry analysis of Western blots performed with an anti-puromycin antibody. Values represent the background corrected sum of lane signal intensity and is the mean of seven biological replicates ± standard deviation. ∗ indicates *p* < 0.05, ∗∗ indicates *p* < 0.005 by paired two-tailed *t* test. *C*, the mean values from (*A*) and (*B*) were plotted as an XY scatter and Pearson correlation analysis was performed revealing a positive correlation between BC200 expression and translation rate as measured by puromycin incorporation. Statistical analysis was performed with GraphPad Prism software. Error bars indicate standard deviation. *D*, representative Western blot of puromycin incorporation assay samples used to generate the data in (*B*). *E*, cell viability measurements by MTT assay time course of stably expression pooled cells and isolated clones assayed at the indicated time points following cell plating. Data were fit to an exponential growth (Malthusian) curve to generate estimated population doubling times. *F*, data from (*A*) and (*E*) were plotted as an XY scatter and Pearson correlation analysis was performed revealing no significant relationship between BC200 expression and rate of cell growth. Error bars indicate standard deviation (expression) and 95% confidence interval (doubling time).

In addition to studying translation rates in stably transfected pooled cells, we also examined the impact of BC200 on translation in isolated single-cell clones by puromycin incorporation and polysome profiles. Isolated clones demonstrated basal BC200 expression levels that varied from unchanged as compared with wildtype MCF-7 to an approximate twofold increase in expression (Fig. 8*A*). Puromycin assays performed on all clones and stable pooled cell lines in parallel demonstrated an elevation in translation levels that exhibited a positive correlation with BC200 expression (Fig. 8, *B*–*D*).

To investigate the observation of reduced growth rates among the BC200 overexpressing clones, we performed MTT assays to monitor cell growth over time. With the exception of clone 11, all BC200-overexpressing clones exhibited an increased doubling time (Fig. 8*E*); however, the degree to which growth was inhibited did not correlate with BC200 expression (Fig. 8*F*). As a second method, polysome profiles were performed on several of the BC200 overexpressing clones that demonstrated results consistent with the puromycin incorporation assays (Fig. S2).

### BC200 comigrates with ribosomal subunits and polysomal RNA

BC200 was previously reported to exist as an 11.4S ribonucleoprotein similar to other expressed Alu RNAs (33, 42). To investigate the mechanism by which BC200 exerts an impact on global translation rates we sought to analyze the migration of BC200 and its identified protein binding partners by sucrose density gradient centrifugation. Consistent with previous reports, we found that BC200 migrates as a single peak clearly separated from the 40S ribosomal subunit (Fig. S3, *A* and *B*). Analysis of four confirmed BC200 protein binding partners revealed that, with the exception of SRP9, they migrated near the top of the gradient and did not associate with ribosomal subunits or actively translating mRNAs (Fig. S3*C*). To increase resolution, gradients were centrifuged for 16 h to separate out the lower-density components. Analysis of fractions taken from extended centrifugation revealed essentially no overlap between BC200 and CSDE1, DHX36, and PABPN1 (Fig. S4, *A* and *B*). The only binding partner comigrating of those tested was SRP9 (Fig. S4*B*).

As these results were inconsistent with an established relationship with BC200, we hypothesized that the conditions to which the cell lysates were subjected were incompatible with maintaining biologically relevant interactions. We therefore tested the impact of formaldehyde cross-linking on migration of BC200 by sucrose density gradient centrifugations. Cross-linking with 0%, 0.5%, 1%, and 2% formaldehyde resulted in a shift of BC200 from the reported 11.4S peak to a broader migration pattern that overlapped both the ribosomal subunits and polysomal RNA (Fig. S5). Increasing formaldehyde concentration progressively preserved BC200 interactions; however, cross-linking with greater than 2% formaldehyde rendered cell lysis inefficient (data not shown). Although 2% formaldehyde is considerably higher than has been used in previous studies, this approach was necessary to maximally preserve BC200 complexes (52, 53).

**Figure 9:**
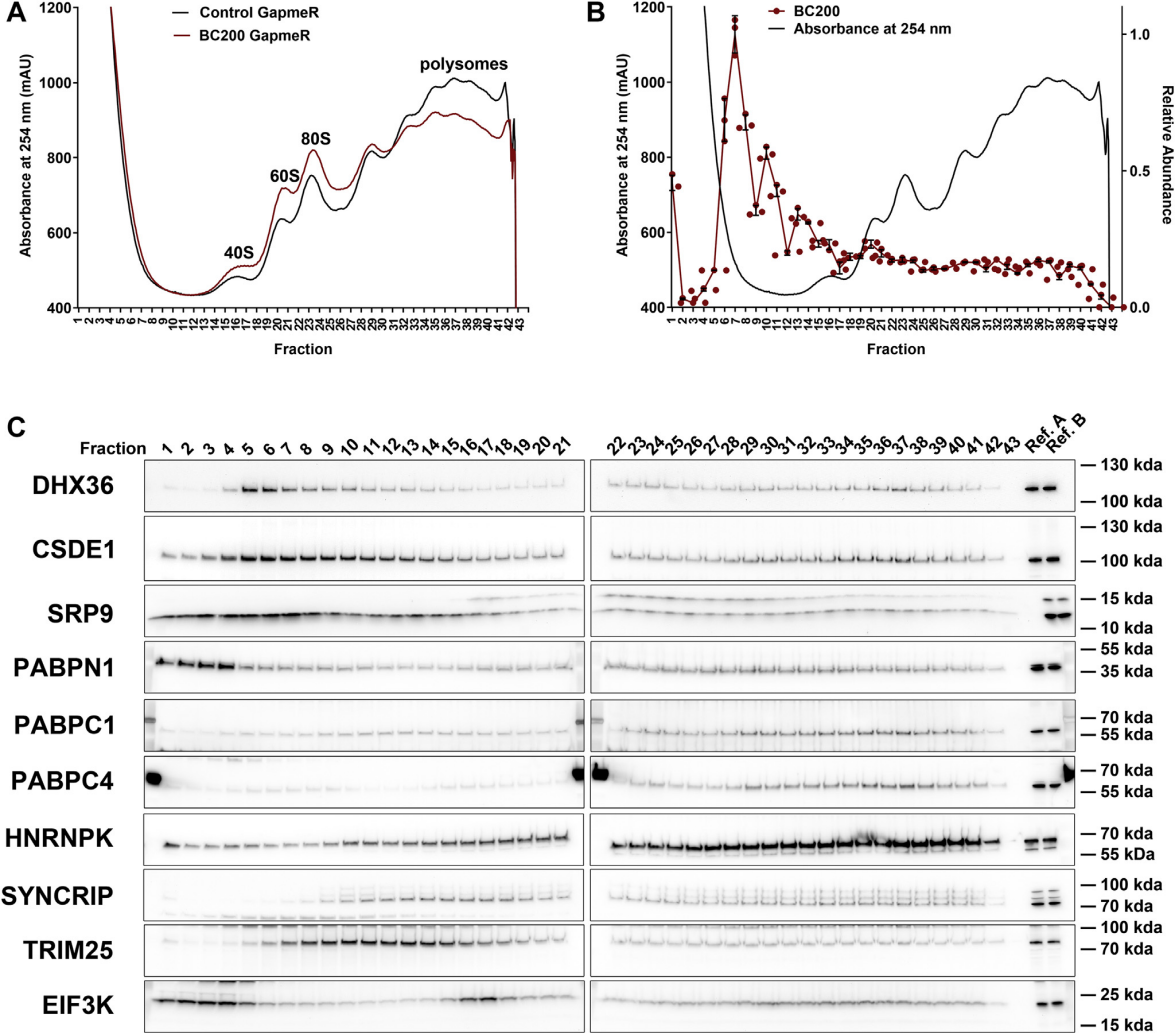
**BC200 migration by cross-linking sucrose density gradient centrifugation.***A*, polysome analysis by sucrose density gradient centrifugation of cell lysates cross-linked with 2% formaldehyde 48 h following transfection of a control (*black*) or BC200 targeting LNA GapmeR (*red*). *B*, fractions were collected from the control profile shown in (*A*) and set aside for RNA and protein analysis. Relative BC200 distribution as measured by RT-qPCR analysis of RNA extracted from the fractions. Line represents the mean of three replicates ± standard deviation. *C*, SDS-PAGE was performed on the fractions described in (*B*). Western blots were performed for the indicated proteins. Exposure was normalized between separate blots with two reference samples (Ref. A and Ref. B) present on each membrane.

Polysome profiles performed under 2% formaldehyde cross-linking revealed absorbance profiles similar to native conditions; however, as expected, cross-linking increased the heterogeneity of the cellular components resulting in less defined peaks. Assignment of 40S, 60S, and 80S peaks was performed by monitoring migration of ribosomal proteins (RPS3, RPL17) and 18S and 28S rRNA in pilot experiments. Consistent with native conditions, knockdown of BC200 was observed to cause a marked reduction in polysomal RNA and elevation in the 40S, 60S, and 80S peaks (Fig. 9*A*). Monitoring of relative BC200 levels throughout the profile revealed comigration with the 40S, 60S, and 80S peaks as well as extended migration through the higher-order polysome fractions (Fig. 9*B*). BC200-binding proteins that were previously completely separated from the BC200-containing fractions now demonstrated similar distribution throughout the profile as BC200 (Fig. 9*C*). Migration of additional BC200-binding proteins HNRNPK, PABPC1, SYNCRIP, PABPC4, and TRIM25 also showed distribution patterns throughout the profile consistent with expectations (Fig. 9*C*). Analysis of the initiation factor EIF3K demonstrated a clear comigration with the 40S subunit indicating that cross-linking conditions maintained biologically relevant interactions and permitted clear differentiation of distinct cellular complexes (Fig. 9*C*).

To confirm an interaction of BC200 with ribosomes under native conditions, BC200 expression constructs containing the Mango-II RNA aptamer inserted in one of three regions of the RNA (insertion after nucleotide 83, 120 and 197) were transfected into HEK-293T cells (Fig. S6, *A*–*D*). The Mango-II aptamer folds into a quadruplex structure that exhibits high binding affinity and specificity for the fluorophore thiazole orange biotin (TO1-biotin) (54, 55, 56). To stabilize the aptamer folding within the 3′ end of BC200, the aptamer was inserted within the context of the F30 scaffold sequence (57). As a negative control, an expression construct was designed to express the F30-Mango-II sequence alone from the U6 snRNA promoter. Forty-eight hours post transfection cells were lysed and pull-downs performed with streptavidin beads preincubated with TO1-biotin. Total RNA stains of the pull-down RNA demonstrate a high enrichment of the aptamer-tagged BC200 as well as the control RNA (Fig. S6*A*). Analysis of coprecipitating proteins revealed specific interaction of known BC200-binding proteins CSDE1 and SRP9 with the aptamer-tagged BC200 but not the control RNA. Confirming the cross-linking sucrose density gradient centrifugations, the ribosomal proteins RPL17 and RPS3 demonstrated enhanced binding to the aptamer-tagged BC200 RNAs as compared with the negative control (Fig. S6*B*).

### BC200 expression is upregulated in response to translation arrest

**Figure 10:**
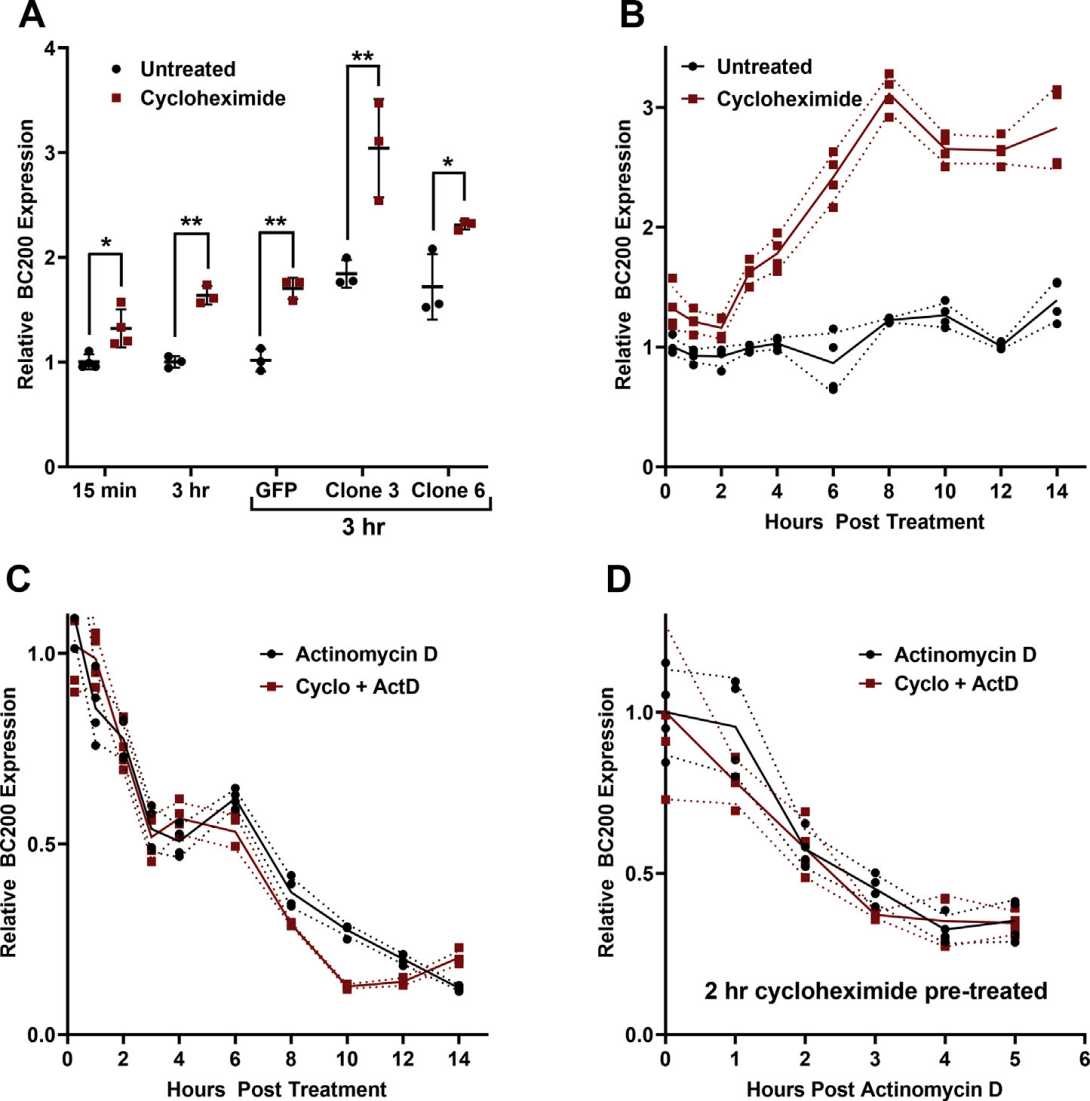
**BC200 is transcriptionally upregulated in response to translational arrest.***A*, relative BC200 expression was measured by RT-qPCR on 25 ng of RNA extracted from wildtype MCF-7 cells or the indicated clones left either untreated or treated with 100 μg/ml cycloheximide. Horizontal line represents the mean of samples measured in triplicate ± standard deviation. ∗ indicates *p* < 0.05, ∗∗ indicates *p* < 0.005 by paired two-tailed *t* test. Cells were treated with cycloheximide for either 15 min or 3 h prior to RNA extraction as indicated. *B*, relative BC200 expression measured by RT-qPCR at the indicated time points following cycloheximide (100 μg/ml) addition to the cell culture media. Line represents the mean of samples measured in triplicate ± standard deviation (*dashed lines*). *C*, as in (*B*), quantification of relative BC200 expression following treatment with Actinomycin D (5 μg/ml) (*black*) or Actinomycin D with cycloheximide (*red*). *D*, as in (*C*); however, Actinomycin D was supplemented 2 h following addition of cycloheximide.

We observed a modest yet consistent elevation in BC200 expression in response to brief cycloheximide pretreatment of cells for polysome profile analyses. BC200 expression was elevated approximately 1.25-fold within 15 min of exposure to cycloheximide and further to 1.75-fold following prolonged (3 h) cycloheximide treatment (Fig. 10*A*). Similar results were observed in control and BC200-overexpressing stable clones (Fig. 10*A*). Time-course studies demonstrated an almost immediate increase in BC200 that was stable for 2 h, followed by a linear increase in expression to approximately threefold basal levels that plateaued at approximately 8 h post treatment. We hypothesized that BC200 turnover in the cell was tied to the rate of translation and therefore treated the cells with either Actinomycin D alone to block transcription and monitor RNA decay or Actinomycin D in combination with cycloheximide to block transcription and translation simultaneously (58). Translation arrest with cycloheximide did not impact the half-life of BC200 significantly (Fig. 10*C*). As the linear increase in BC200 begins 2 h post cycloheximide treatment (Fig. 10*B*), we repeated the RNA decay measurements but delayed the addition of Actinomycin D until 2 h post cycloheximide. In this case as well, the decay of BC200 was unchanged by cycloheximide pretreatment (Fig. 10*D*). As half-life was unchanged by cycloheximide, we concluded that the elevation in BC200 expression was due to the transcriptional upregulation of the BC200 gene.

## Discussion

The importance of noncoding RNA in diverse cellular processes and disease states continues to become increasingly evident (59, 60). Alu RNAs are emerging as key regulatory elements both in the context of Alu insertions within transcribed noncoding regions of mRNAs as well as independently as discrete RNA polymerase III transcripts (1, 3, 4, 5, 33, 35, 39, 40, 41). The sheer abundance and high degree of sequence similarity between Alu transcripts and the difficulty in discerning RNA polymerase III transcripts from “passenger” Alu sequences within larger mRNAs adds a significant level of complexity in discerning the role these RNAs play in the context of cell physiology (1). BC200 is a highly expressed and functional Alu-containing RNA that has a clear neuronal role and dysregulation in cancer (61). From both a disease perspective and in gaining a clearer understanding of the normal function of this expansive class of noncoding RNAs, the study of BC200 function is of great value. The fact that in rodents an ancestrally unrelated transcribed repetitive element (BC1) appears to share a similar or identical function to BC200 and that a transcribed Alu insertion is observed in prosimian primates at the identical genomic location as BC200 both provides further research tools and adds significance to the pursuit of understanding BC200 function.

Despite having been initially described as a brain-specific transcript over 30 years ago, a comprehensive understanding of BC200 function has remained elusive (8). An emerging theme in the study of BC200 has been its role as a translational regulator, specifically as a general repressor of global translation rates (32). As all of the studies to date have looked at overexpression of BC200 through transfection of *in vitro* transcribed RNA into cultured cells or supplementation of BC200 to *in vitro* translation assays, we wished to evaluate the impact of BC200 knockdown on translation levels in a cellular context. Our data clearly indicate that BC200 knockdown is correlated with a dramatic reduction in global translation rates using multiple methods and in a wide-range of cell lines (Fig. 1, Fig. S1). In replicating some of the previously used methodology, we determined that transfection of *in vitro* transcribed RNAs produces aberrant results owing to the triggering of an innate immune response (Fig. 2). Although BC200, BC1, and a scrambled mutant of BC200 all significantly repressed mRNA translation, the BC200 truncation BC119 had a modest translation stimulatory effect. This was despite the fact that all transfections elicited an innate immune response as was indicated by RIG-I induction. One explanation for this is the reported ability of Alu RNAs to repress PKR activity (41). It is possible that the non-Alu sequence at the 3′ end of BC200 impairs this function, which is observed with the Alu-only truncation BC119.

To avoid triggering an innate immune response with exogenous RNA, we attempted to study BC200 function by overexpression from transfected plasmid DNA. Here we also observed a substantial impact on translation rates from control transfections indicating that both transient RNA and plasmid transfections are not suitable for the study of mechanisms relevant to mRNA translation rates (Fig. 4). Abandoning transient expression methodologies, we next developed cell lines in which the control and RNA-expressing plasmids were stably incorporated into the genome of the host cell. Overexpression levels were modest, not exceeding 2-fold of wildtype, whereas transient expression produced robust levels (50-fold) of the encoded RNAs. This is likely due to epigenetic silencing of the internal RNA polymerase III promoter (3). Despite only modest increases in BC200, we observed a clear elevation in higher-order polysomes within the stably transfected pool as compared with the control plasmid. Interestingly, mutation of the unique sequence at the 3′ end of the RNA appeared to impair function with a reduction in higher-order polysomes observed (Fig. 5*C*). This was not consistent in puromycin assays performed subsequently (Fig. 8*B*), possibly due to compensatory selection within the stable pooled cells over time. Although the G22 RNA produced similar results to BC200, G22 expression was not able to compensate for BC200 under the context of BC200 knockdown. This was likely due to the reduction in G22 expression that was also observed upon BC200 knockdown. Although the region targeted in BC200 varies considerably in G22 RNA, the LNA GapmeR is still able to potentially make nine base-pairing interactions (Fig. 7*E*). The simplest explanation is that G22 expression is being knocked down through off-target effects of the BC200 GapmeR. BCMUT was not expected to be able to compensate for BC200 as the stable pooled cells did not demonstrate an elevation in higher-order polysomes indicating the introduced mutations abrogated function. Interestingly, BCMUT expression was significantly upregulated upon BC200 knockdown, which may indicate an attempt by the cells to counterbalance BC200 knockdown by transcriptionally upregulating the BC200 gene and/or other Alu RNAs (Fig. 7*C*).

As the stable BC200 overexpression pool would be prone to selection for faster growing clones, we isolated single-cell clones to preserve any phenotype that would be selected against in the pooled cells. Single-cell clones exhibited variable levels of BC200 but expression was generally higher than that of the pooled cells. A decreased growth rate was observed for both the pooled BC200-overexpressing cells and the isolated single-cell clones, indicating that elevated BC200 expression may be selected against over time. Although all the BC200-overexpressing clones had a longer doubling time than the wildtype MCF-7 cells, there was no significant correlation between the degree to which BC200 was expressed and the rate of cell growth (Fig. 8*F*). This contrasts with the global translation rate measurements, in which we did observe a positive correlation between BC200 expression levels and translation rates as measured by puromycin incorporation (Fig. 8*C*). These data present a paradox as overexpression of BC200 in tumor cells is correlated with poor prognosis and BC200 knockdown in cell lines impairs cell growth. Although it may seem inconsistent to reconcile this with a reduced growth rate observed in BC200 overexpressing cells, it is possible that the reduced growth rate is related to the site of plasmid integration into the host cells genome. An additional possibility is that BC200 has a growth inhibitory effect while at the same time is required at a minimal level to maintain cell viability.

Both BC200 and other transcribed Alu RNAs have been reported to exist as discrete ∼11S RNPs bound to SRP9/14 and not associated with ribosomal subunits or actively translating mRNAs (33, 42). We replicated similar results to this by monitoring BC200 migration through standard polysome profiles. Western blotting for BC200 binding partners, however, called these data into question, as prolonged separation by ultracentrifugation revealed that BC200 did not comigrate with many of its previously confirmed protein interaction partners (Figs. S3 and S4). To assess whether biologically relevant complexes are not persisting under the conditions of sucrose density gradient centrifugation we cross-linked the cells with formaldehyde prior to cell lysis. Formaldehyde cross-linking resulted in a substantial subpopulation of BC200 that migrated throughout the gradient along with ribosomal subunits as well as actively translating mRNAs. The proportion of BC200 interacting with ribosomes or ribosome-bound mRNAs may be considerably higher than observed owing to incomplete cross-linking; however, excessive cross-linking impairs cell lysis and may preserve more distant interactions. Therefore, although it is difficult to precisely conclude the proportion of BC200 that comigrates with ribosomal subunits and polysomal RNA, the data presented suggest that BC200 may in fact be exerting its function through these interactions and warrants further investigation to gain a clearer understanding of how BC200 elevates translation rates.

Finally, we followed up on an observation that cycloheximide treatment was causing consistent increases in BC200 expression in cells prepared for polysome profiling. Cycloheximide treatment caused a nearly immediate elevation in BC200 to approximately 1.25-fold, which remained steady for 2 h at which point expression steadily increased until it reached 3-fold at 8 h (Fig. 10). A literature search revealed that a similar response has been observed with other transcribed Alu RNAs in response to both cycloheximide as well as other translation inhibitors (33, 35). The most straightforward explanation of this is that the cells respond to translational stress by upregulating RNAs that may stimulate mRNA translation to restore protein production. This supports our proposed function of BC200 as a translational activator.

Here, for the first time we present data that the function of BC200 is to stimulate translation, either globally or at a subset of target mRNAs. Although these data disagree with the previously proposed function of BC200 as a translation repressor, it is consistent with some reports on the function of other generic Alu transcripts (33, 35, 40, 41). Further work is needed to discern the molecular mechanism by which this is accomplished and determine whether there is any specificity toward the transcripts affected. The identification of aberrant results caused by an innate immune response from transient transfections of *in vitro* transcribed RNAs or plasmids should serve as a discouragement of these methods in favor of stable overexpression or inducible constructs when attempting to interpret the function of other noncoding RNAs. We are currently developing stable inducible cell lines for BC200 expression as well as aptamer tagged BC200 expression constructs, which we hope will aid in answering these questions and further shed light on the function of this noncoding RNA.

## Experimental procedures

### Cell culture and reagents

The HEK293T cell line was a gift from Dr Thomas Klonisch; the MCF-7, SK-BR-3, T-47D, A549, and MDA-MB-231 cell lines were a gift from Dr Spencer Gibson; the SK-OV-3 cell line was a gift from Dr Peter Pelka; the A375 cell line was a gift from Dr Jens Kurreck. Cell culture conditions were as previously published (17). DNA primers and LNA GapmeRs were purchased from Integrated DNA Technologies (Coralville, IA). All standard laboratory chemicals and reagents were purchased from Thermo Fisher Scientific (Ottawa, Canada).

### LNA GapmeR and plasmid transfection

LNA GapmeRs were transfected using Lipofectamine RNAiMax (Thermo Fisher Scientific) according to the manufacturer’s protocol. Reverse transfections were performed by combining 100 pmole of GapmeR with 7.5 μl Lipofectamine RNAiMax in 250 μl Opti-MEM media (Thermo Fisher Scientific) per well of a 6-well plate. Two milliliters of cell suspension was added such that cells were approximately 80% confluent 48 h post transfection. The following seeding cell densities were used: MCF-7 at 650,000 cells per ml, HEK-293T at 800,000 cells per ml, SK-BR-3 at 1,000,000 cells per ml, SK-OV-3 at 300,000 cells per ml, A549 at 400,000 cells per ml, MDA-MB-231 at 500,000 cells per mL, T-47D at 650,000 cells per ml, HeLa at 400,000 cells per ml, and A375 at 350,000 cells per ml. Transfections into other cell culture plates were scaled accordingly by volume. The BC200 targeting GapmeR sequence is as follows: +A∗+G∗+G∗G∗A∗A∗G∗T∗T∗A∗C∗G∗C∗+T∗+T∗+A. The Negative Control GapmeR sequence is as follows: +A∗+A∗+C∗A∗C∗G∗T∗C∗T∗A∗T∗A∗+C∗+G∗+C. “+N” indicates Affinity Plus (locked nucleic acid) bases and “N∗” indicates phosphorothioated DNA bases. For control transfections, the cDNA sequence of enhanced green fluorescent protein was cloned into the HindIII and XhoI sites of the pCDNA3.1 plasmid. Expression cassettes for BC200 (−32 to +224), BCMUT (−32 to +224), and G22 (−383 to +359) were cloned upstream of the CMV promoter driving GFP expression using the BglII and MluI sites of the pCDNA3.1-GFP plasmid using standard molecular biology techniques. The BCMUT plasmid contains the identical sequence as BC200 except for the scrambling of nucleotides within the GapmeR targeting region (159–174). Plasmid transfections were performed using Turbofect transfection reagent according to the manufacturer’s protocol (Thermo Fisher Scientific). Stably expressing cells were selected with G418 (300 μg/ml) and single-cell clones were isolated by performing limiting dilutions of stable pooled cells into 96-well plates.

### *In vitro* transcription, RNA purification, and transfections

*In vitro* transcription and purification of BC200, BC1, BCSCR, and BC119 was performed as previously described (62). Following transcription, *in vitro* transcribed RNAs were purified by phenol-chloroform extraction followed by size exclusion chromatography and subsequently filter sterilized by passage through a 0.22-μm syringe filter. Plasmids containing RNA sequence with a 5′ T7 promoter and 3′ linearization site were synthesized by Genscript Inc (Piscataway, NJ, USA). The sequence of BCSCR has been reported previously (34). RNA transfections were performed with Lipofectamine RNAiMax according to the manufacturer’s protocol (Thermo Fisher Scientific). For puromycin incorporation assays, 10 pmole of RNA was used per well of a 6-well dish with 7.5 μl of Lipofectamine RNAiMax. For polysome profiles, 120 pmole of RNA was used per 150-mm tissue culture dish with 90 μl Lipofectamine RNAiMax. Translation rates were assayed by both methods 14 h post transfection.

### Measurement of translation by puromycin incorporation assay

mRNA translation rates were assessed by measurement of puromycin incorporation using the previously described SUnSET assay with minor modifications (45). Following indicated treatments, cell culture media was replaced with fresh prewarmed media containing 10 μg/ml puromycin. Cells were incubated for 5 min at 37 °C following which media was aspirated and replaced with PBS containing 100 μg/ml cycloheximide. PBS was aspirated and replaced with 0.05% trypsin containing 100 μg/ml cycloheximide. Cells were incubated 3 min and resuspended by adding an equal volume of cell culture media containing 100 μg/ml cycloheximide. Cells were transferred to 1.5-ml microcentrifuge tubes and placed on ice. Cells were pelleted and washed with PBS containing 100 μg/ml cycloheximide. Cell pellets were resuspended in RIPA buffer containing 1X HALT protease and phosphatase inhibitor cocktail (Thermo Fisher Scientific). Protein concentration was assessed by the standard Bradford assay, and protein lysates were subjected to SDS-PAGE and Western blotting. A minimum of three biological replicates was performed per condition, and relative puromycin incorporation was measured by densitometry analysis of the background corrected sum of lane signal intensity (AlphaView software).

### SDS/PAGE, Western blotting, and antibodies

SDS/PAGE and Western blotting were performed as previously described (63). The following antibodies were purchased from Thermo Fisher Scientific: Mouse anti-Puromycin (MABE343MI), Rabbit anti-Actin (PIPA1183), Mouse anti-SYNCRIP (MAB11004MI). The following antibodies were purchased from Abcam (Toronto, ON, Canada): Rabbit anti-PABP (ab21060), Rabbit anti-CSDE1 (ab201688), Rabbit anti-TRIM25 (ab167154), Rabbit anti-PABPC4 (ab220832, Abcam), Rabbit anti-PABPN1 (ab75855), Mouse anti-HRNPNK (ab39975). The following antibodies were purchased from Proteintech (Rosemont, IL, USA): Rabbit anti-SRP9 (11195-1-AP), Rabbit anti-SRP14 (11528-1-AP), Rabbit anti-STRAP (18277-1-AP). The following additional antibodies were used: Mouse anti-Tubulin (T6074, Sigma-Aldrich), Rabbit anti-RIG-I (3743S, Cell Signaling Technologies, Danvers, MA, USA), Rabbit anti-EIF3K (CSB-PA866204ESR1HU, CusaBio), and Mouse anti-DHX36 (Clone 12F33, made in-house).

### Polysome profiling and Protein/RNA analysis of sucrose density gradients

Sucrose gradients were prepared in 20 mM Hepes pH 7.6, 100 mM KCl, 5 mM MgCl_2_ containing 100 μg/ml cycloheximide, 1x HALT protease and phosphatase inhibitor cocktail and Ribolock RNAse inhibitor (Thermo Fisher Scientific). Gradients ranging from 10% to 50% sucrose were prepared by overlaying 3 ml of each sucrose-containing buffer in 5% sucrose increments with flash freezing at −80 °C between each layer in Beckman Coulter Ultra-Clear 25 x 89 mm ultracentrifuge tubes (Thermo Fisher Scientific). Gradients were frozen at −80 °C and thawed by placing at 4 °C overnight prior to the day of experiment. On the day of experiment, cells from an approximately 80% confluent 150-mm dish were treated with 100 μg/ml cycloheximide for 5 min, then pelleted and washed twice with 10 ml cold PBS containing 100 μg/ml cycloheximide. The cell pellet was resuspended in hypotonic buffer (5 mM Tris-Cl pH 7.5, 2.5 mM MgCl_2_, 1.5 mM KCl, 1x HALT protease, and phosphatase inhibitor cocktail). After resuspension, the following components were added: 5 μl 10 mg/ml cycloheximide, 1 μl 1M DTT, 2 μl Ribolock. The cell suspension was vortexed for 5 s, following which 25 μl of 10% Triton-X 100 and 25 μl of sodium deoxycholate was added followed by an additional 5 s of vortexing. The cell lysate was centrifuged at maximum speed for 10 min, and the supernatant was transferred to a Corning Spin-X 0.45-μm centrifugal filter. The lysate was centrifuged 15 min to pass through the filter and transferred to a new tube on ice. Lysates were normalized for total protein content and an equal volume of lysate from each sample was overlaid on the top of the gradients. Gradients were balanced and centrifuged for 2.5 h at 32,000 rpm on a Beckman Coulter L8-80 preparative ultracentrifuge using a SW32 swinging bucket rotor. Following centrifugation, a 6-inch 20-gauge blunt-ended stainless-steel needle was carefully inserted through the center of the gradient to the bottom of the tube. Using a peristaltic pump in line with a column valve, the gradient was pumped into an AKTA Purifier 10 FPLC connected to a Frac-950 fraction collector (GE Life Sciences) at a flow rate of 1.5 ml/min with the AKTA Purifier pumping water into a waste container at an equal flow rate. Inline absorbance at 254 nm was monitored and fractions collected for further RNA and protein analyses. For RNA analysis, fractionated RNA was purified using the GeneJet RNA Clean-up and concentration micro kit (Thermo Fisher Scientific). For protein analysis, fractions were combined with 5x SDS loading dye for SDS-PAGE and Western blotting.

### Cross-linking sucrose density gradient centrifugation

For cross-linking sucrose density gradients, two 80% confluent 150-mm dishes were used per condition. Following cycloheximide treatment, cell culture media was replaced with serum-free Dulbecco's modified Eagle's medium containing 100 μg/ml cycloheximide and 2% formaldehyde (diluted from 16% methanol-free formaldehyde). Plates were rocked for 10 min at room temperature at which point glycine was added to a final concentration of 100 mM. Lysis was performed as described above, with the added step of five repeats of sonication for 10 s each at 30% output on ice with a Branson model 150T Sonic Dismembrator fitted with a microtip (Thermo Fisher Scientific). Following sonication, lysates were centrifuged at maximum speed for 10 min on a refrigerated table top centrifuge and the above-described protocol was followed for sucrose density gradient centrifugation.

For protein analysis, samples were heated for 15 min at 95 °C to reverse cross-links prior to SDS-PAGE. For RNA extraction, 400 μl of each fraction was combined with 200 μl 3x reverse cross-linking buffer (3x PBS, 6% N-lauroyl sarcosine, 30 mM EDTA, 5 mM DTT) and 20 μl Proteinase K (20 mg/ml). Samples were incubated for 1 h at 42 °C with shaking followed by 1 h at 55 °C with shaking. Following cross-link reversal and protein digestion, RNA was purified with the GeneJet RNA Clean-up and Concentration Micro kit according to the manufacturer’s protocol (Thermo Fisher Scientific).

### Pull-down assays of Mango-II tagged BC200

Expression constructs consisting of the BC200 RNA with insertions of the Mango-II aptamer were synthesized by Integrated DNA Technologies. BC200-Mango RNAs were expressed from the BC200 minimal promoter sequence and a control RNA consisting of the Mango-II aptamer alone inserted into the F30 scaffold sequence was expressed from the U6 snRNA promoter. Cells were plated into 150-mm dishes and transfected as described above. Forty-eight hours post transfection cells were treated with cycloheximide for 10 min and then lysed in 250 μl cytoplasmic lysis buffer per 150-mm dish (25 mM Hepes, pH 7.9, 5 mM KCl, 0.5 mM MgCl_2_, 0.5% (v/v) NP-40) supplemented with protease and RNase inhibitors (Halt protease and phosphatase inhibitor cocktail and Ribolock RNase inhibitor [Thermo Fisher Scientific]) followed by a 5-min incubation at 4 °C with end-over-end mixing. Following incubation, the volume was increased to 500 μl and buffer composition was adjusted to 25 mM Hepes pH 7.9, 100 mM KCl, 0.5 mM MgCl_2_, 0.25% (v/v) NP-40 (KCl IP Buffer). Insoluble material was removed by centrifugation at 14,000 rpm for 10 min in a bench top microcentrifuge at 4 °C. Protein concentration was assessed by the standard Bradford assay, and all lysate concentrations were normalized to a protein concentration of 3 mg/ml. Five hundred microliters of lysate was used per immunoprecipitation. Streptavidin magnetic beads were pre-equilibrated with TO1-biotin by adding 10 μl TO1-biotin (667 μM) per 100 μl of beads and incubating with end-over-end mixing for 30 min. Unbound TO1-biotin was removed by washing the beads 3-fold in KCl IP Buffer. To capture BC200-Mango RNP complexes, 50 μl of beads was added to the lysate followed by end-over-end mixing at 4 °C for 1 h. Following incubation, beads were washed fourfold in KCl IP Buffer and then resuspended in 1X SDS load dye for SDS-PAGE and Western blotting or RNA was extracted from the beads using the RNA Clean-up and concentration micro kit (Thermo Fisher Scientific).

### RNA quantification by RT-qPCR

RT-qPCR analysis was performed using an Applied Biosystems StepOnePlus instrument with the RNA to Ct One-step RT-qPCR kit (Thermo Fisher Scientific). Reverse transcription and cycling parameters were carried out as per the manufacturer’s specifications (Thermo Fisher Scientific). Twenty-five nanograms of template RNA was used in all RT-qPCR reactions. Reaction specificity was confirmed by melt-curve analysis as well as agarose gel electrophoresis of reaction products. A minimum of three independent experiments were performed for each sample and measured in triplicate. The following primers were used: BC200-forward, ATAGCTTGAGCCCAGGAGTT; BC200-reverse, GCTTTGAGGGAAGTTACGCTTAT; GAPDH-forward, ACCCACTCCTCCACCTTTG; GAPDH-reverse, CTCTTGTGCTCTTGCTGGG; G22-forward, GAGTCTGAGGTTGCAGTGG; G22-reverse, CGC ACAGTTGCCTTGTTT; BCMUT-forward, CTGGGCAATATAGCGAGACC; BCMUT-reverse, TTGCTAGTGTCACGATGGTATG.

### Cell viability assays

Cell viability was assessed using the MTT assay as described previously (64). Cell doubling time was estimated by fitting viability assay time course data to an exponential (Malthusian) growth equation (Y = Y0∗exp(k∗x)) where Y0 is the starting population, k is the rate constant, x is time, and doubling time is calculated as ln(2)/k using GraphPad Prism software.

## Data availability

All of the data are contained within the article.

## Conflict of interest

The authors declare that they have no conflicts of interest with the contents of this article.
