# Supplementary material for: The noncoding RNA BC200 associates with polysomes to positively regulate mRNA translation in tumor cells
Source: J Biol Chem. 2020 Nov 24;296:100036. doi: 10.1074/jbc.RA120.015775 (PMC7949042; doi:10.1074/jbc.RA120.015775)
Supplement: Figs S1–S6 [file mmc1.pdf]

The non-coding RNA BC200 associates with polysomes to positively regulate mRNA translation in tumour cells

Evan P. Booy, Daniel Gussakovsky, Taegi Choi and Sean A. McKenna

Supporting Figures 1-6

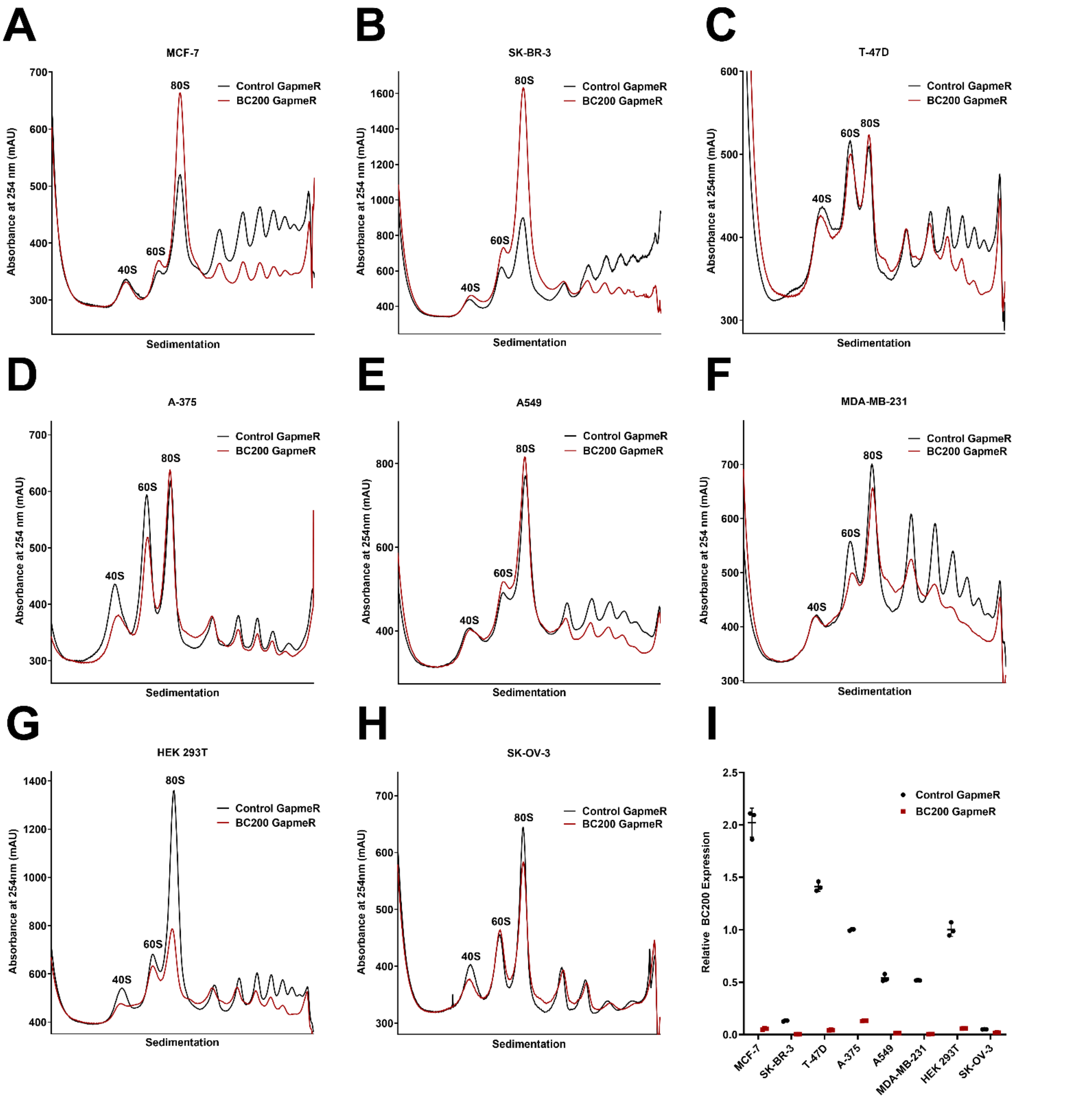

Supplementary Figure 1. BC200 knock-down inhibits translation. Polysome analysis by sucrose density gradient centrifugation of cell lysates 48 hour following transfection of a control (black) or BC200 targeting LNA GapmeR (red) in **(A)** MCF-7, **(B)** SK-BR-3, **(C)** T-47D, **(D)** A-375, **(E)** A549, **(F)** MDA-MB-231, **(G)** HEK-293T, and **(H)** SK-OV-3 cells. **(I)** RT-qPCR analysis of relative BC200 expression 48 hours following transfection of a non-targeting control GapmeR (black circles) as compared to a BC200 targeting GapmeR (red squares) in the indicated cell lines. Horizontal line represents the mean of samples measured in triplicate +/- standard deviation.

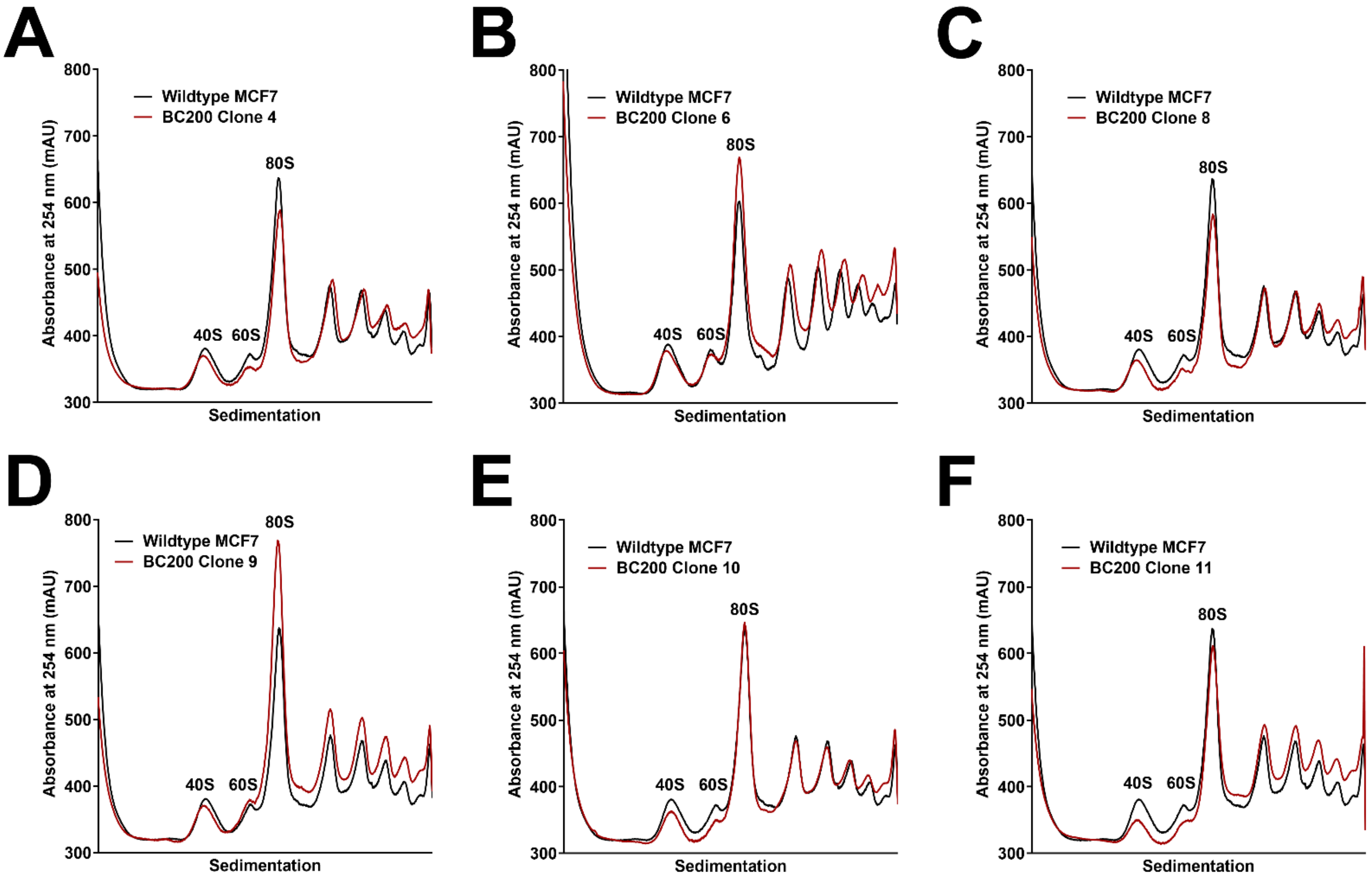

Supplementary Figure 2. BC200 over-expression enhances translation. (A-F) Polysome analysis by sucrose density gradient centrifugation of cell lysates 48 hour following plating of wild-type MCF-7 (black) or the indicated BC200 over-expression clones (red).

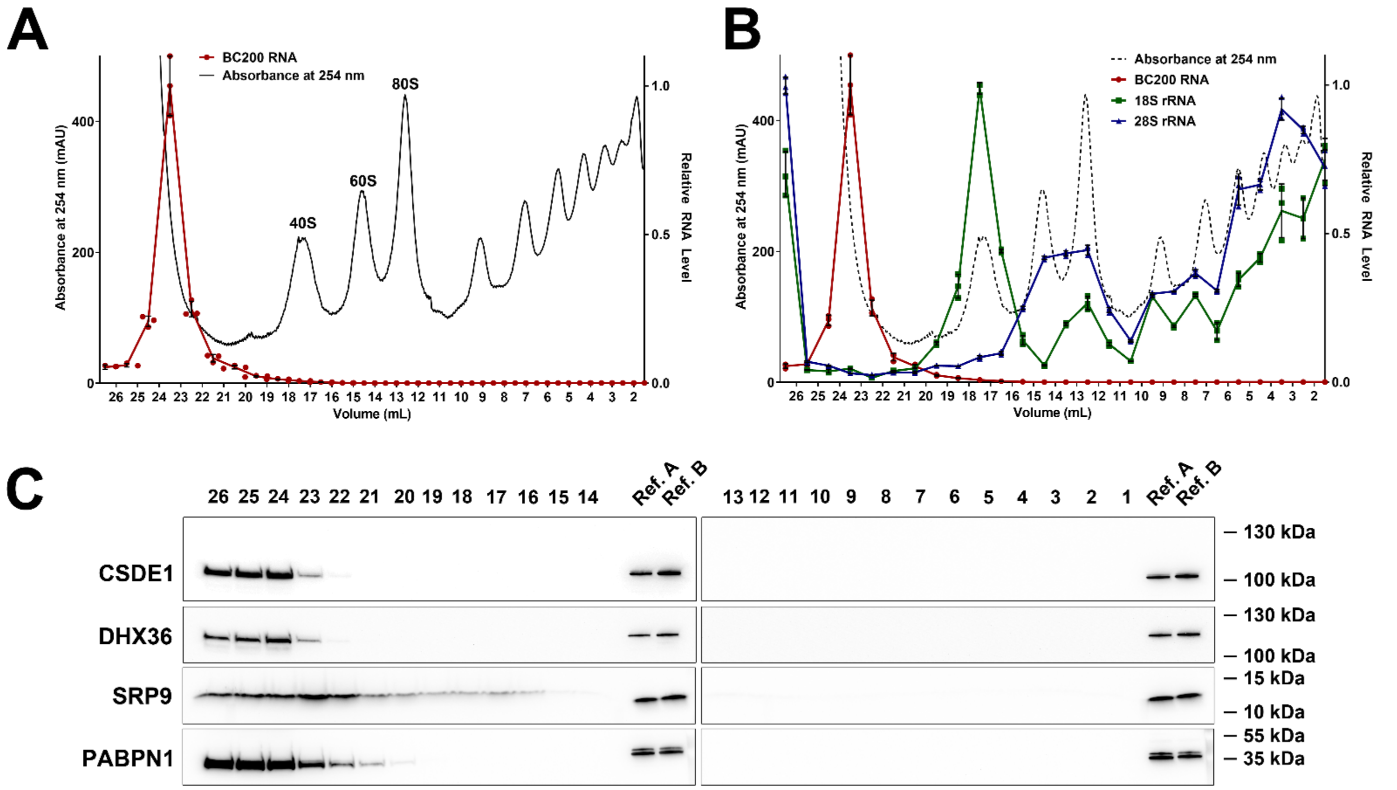

Supplementary Figure 3. BC200 and its binding partners migrate as a low density particle by sucrose density gradient centrifugation. (A) Relative BC200 distribution (red) as measured by RT-qPCR analysis of RNA extracted from fractions collected from the overlaid polysome profile (black). Data represents the mean of three replicates +/- standard deviation. **(B)** Relative distribution of the 18S (green) and 28S (blue) rRNAs as measured by RT-qPCR analysis as in (A) overlaid with BC200 distribution (red) and absorbance profile (black dashed line). Data represents the mean of three replicates +/- standard deviation. **(C)** Western blots of indicated proteins from the fractions collected from the control profile shown in (A). Data was normalized between separate blots with two reference samples (Ref. A and Ref. B) present on each membrane.

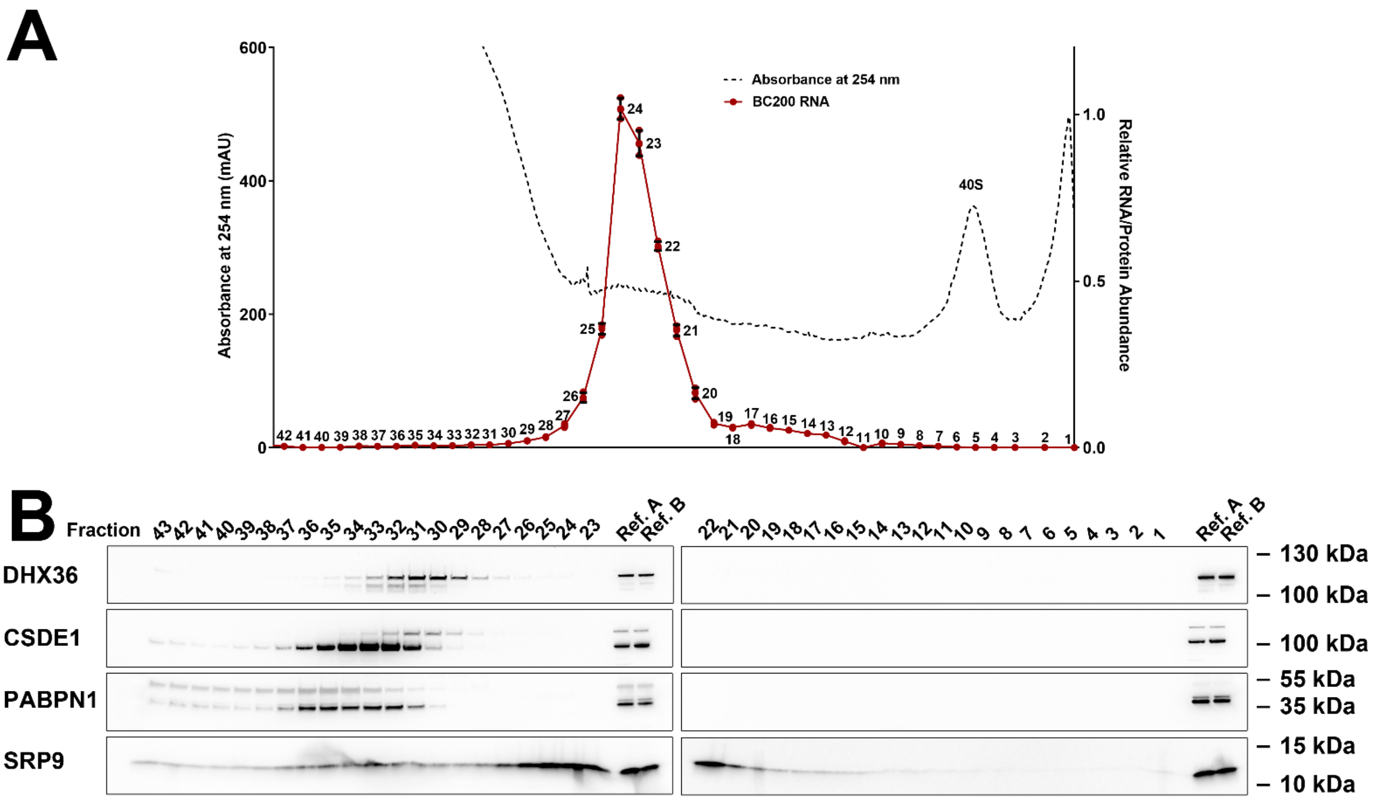

Supplementary Figure 4. BC200 migration following extended (16 hr) ultracentrifugation. (A) Relative BC200 distribution (red) as measured by RT-qPCR analysis of RNA extracted from fractions collected from the overlaid polysome profile (black dashed line). Data represents the mean of three replicates +/- standard deviation. **(B)** Western blots of indicated proteins from fractions collected in (A). Data was normalized between separate blots with two reference samples (Ref. A and Ref. B) present on each membrane.

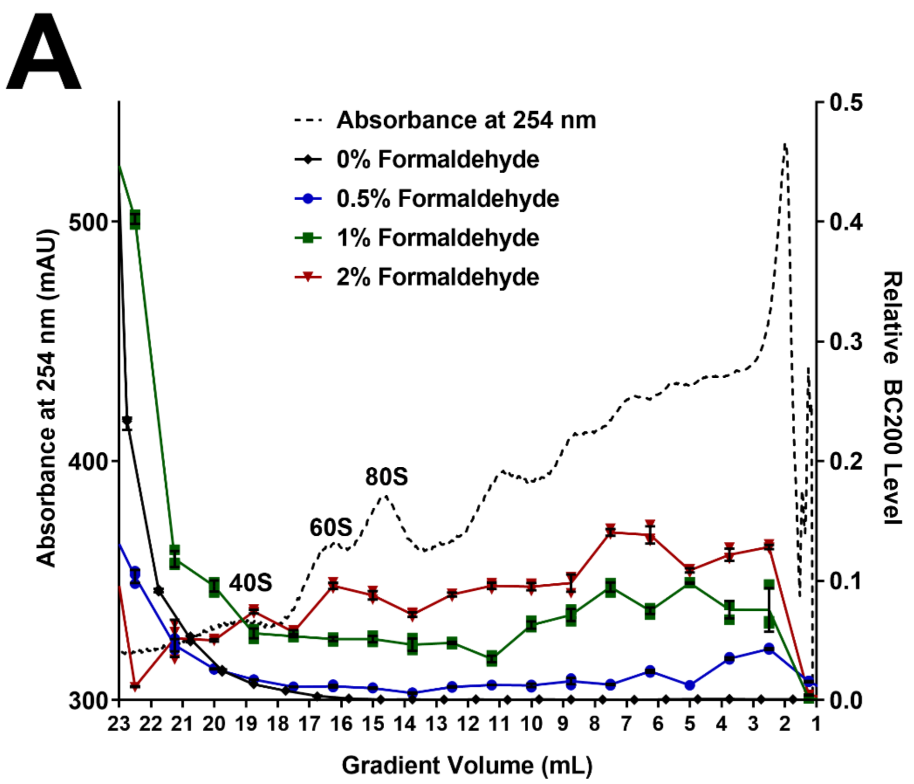

Supplementary Figure 5. BC200 migration with crosslinking using variable formaldehyde concentrations. (A) Relative BC200 distribution as measured by RT-qPCR analysis of RNA extracted from fractions collected from polysome profiles performed under 0% (black), 0.5% (blue), 1% (green) and 2% (red) formaldehyde. The polysome profile from the 2% formaldehyde condition is shown as a black dashed line. Data represents samples measured in triplicate +/- standard deviation.

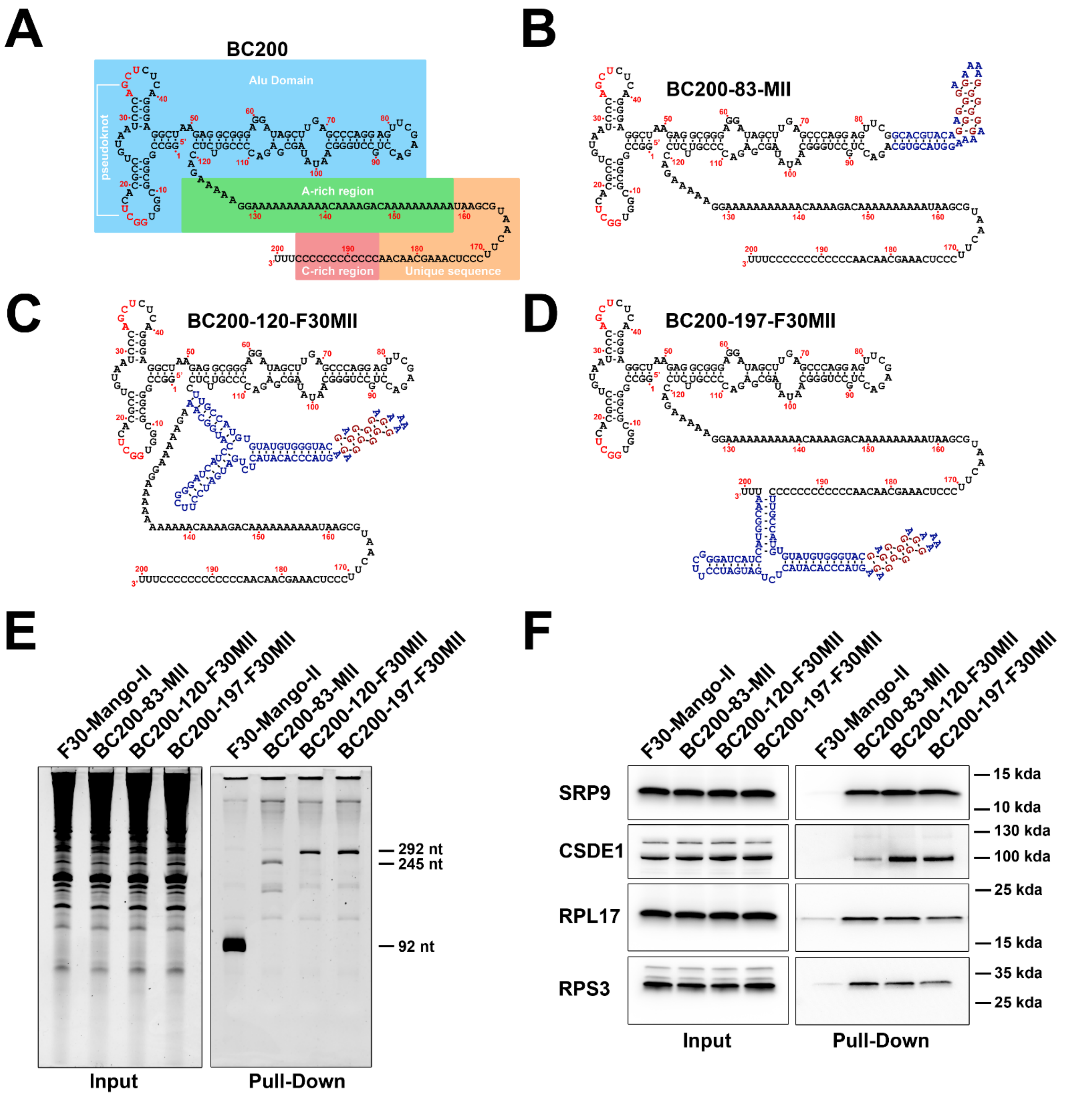

Supplementary Figure 6. BC200-Mango-II pull-downs confirm interactions with ribosomal proteins. (A) BC200 sequence and predicted secondary structure. (B) Sequence and predicted secondary structure of the BC200-83-MII RNA. (C) Sequence and predicted secondary structure of the BC200-120-F30MII RNA. (D) Sequence and predicted secondary structure of the BC200-197-F30MII RNA. (E) Panel 1: Denaturing TBE-UREA gel electrophoresis of 1 µg RNA extracted from the lysates of HEK-293T cells transfected with the indicated expression constructs and stained with SYBR Gold total RNA stain. Panel 2: Denaturing TBE-UREA gel electrophoresis of 10% of the RNA extracted from TO1-Biotin conjugated streptavidin magnetic beads following pull-down of RNP complexes from cell lysate. (F) Western blot of proteins extracted from the BC200-Mango II pull-downs shown in (E).
